# miR-139-5p activates ferroptosis by inhibiting the expression of HMG-CoA reductase to inhibit the progression of glioma

**DOI:** 10.1038/s41420-025-02532-7

**Published:** 2025-05-21

**Authors:** Zhongsheng You, Fei Wu, Yaofeng Zheng, Hongling Yang, Jianbo Ye, Hongyi Cai, Chuangcai Luo, Yang Liu, Yiquan Ke, Xiangdong Xu

**Affiliations:** 1 Department of Neuro-oncological Surgery, Zhujiang Hospital, Southern Medical University, Guangzhou, PR China; 2 The Neurosurgery Institute of Guangdong Province, Zhujiang Hospital, Southern Medical University, Guangzhou, PR China; 3 Department of Ultrasound Diagnosis, Daping Hospital, Army Medical University, Chongqing, China

**Keywords:** CNS cancer, miRNAs

## Abstract

Glioma is the most aggressive and common tumour in the central nervous system. It has been reported that miR-139-5p plays an important role in regulating tumour progression. However, whether miR-139-5p affects the progression of glioma and the specific mechanism remains to be explored. Through experiments involving down-regulation or overexpression of miR-139-5p and treatment with simvastatin (SIM), qRT-PCR and Western Blot were used to detect the expression levels of related genes. Transmission electron microscopy (TEM) and corresponding kits were used to detect the changes in ferroptosis and cholesterol content in glioma cells. RNA-seq analysis was used to explore the specific mechanism by which miR-139-5p regulates ferroptosis. Our results demonstrate that miR-139-5p expression is significantly reduced in glioma cells compared to normal glial cells and is associated with poor prognosis. Overexpression of miR-139-5p promotes ferroptosis and inhibits tumour cell proliferation by downregulating HMG-CoA reductase (HMGCR) expression, consequently hindering glioma progression. Additionally, we found a synergistic effect between miR-139-5p overexpression and SIM treatment in promoting ferroptosis in gliomas. These findings suggest that miR-139-5p could serve as a potential therapeutic target for glioma treatment, particularly in combination with SIM. This study demonstrated that miR-139-5p promoted ferroptosis in glioma cells by down-regulating HMGCR expression and cholesterol synthesis. Moreover, miR-139-5p and SIM had a synergistic effect in promoting ferroptosis to prevent glioma progression.

## Introduction

Gliomas represent the most frequent primary intracranial malignant tumours, accounting for approximately 50% of all primary intracranial central nervous system tumours. Their main biological characteristics include high mortality, high recurrence rates, uncontrolled invasive growth, and strong angiogenic ability. In low-grade gliomas (grades I and II), these features are less pronounced, and the prognosis is relatively favorable. However, the prognosis of grade III and IV gliomas is poor, particularly grade IV IDH wild-type glioblastoma, which has the worst prognosis and the shortest survival [1, 2]. Although substantial progress has been made in treating malignant brain gliomas through combined surgical resection, postoperative radiotherapy and chemotherapy, immunotherapy, and vascular-targeted therapy, current treatments could not control tumour growth. Therefore, there is an urgent need to find effective targets for diagnosis and treatment of glioma [3].

Ferroptosis is a form of cell death that differs from apoptosis, necrosis, or autophagy. It was first proposed by Stockwell in 2012 [4] and depends on iron and lipid peroxidation. Studies have reported that ferroptosis can be activated by various physiological and pathological stimuli. It clears cells that either lack nutrients in the internal environment or have been destroyed by infection or peripheral stimulation. Ferroptosis plays a significant role in inhibiting development of tumours [5, 6]. Cancer cells are under constant oxidative stress and have a delicate balance between thiols and catalysed iron. Ferroptosis is not a common occurrence during cancer development and its specific molecular mechanisms require further study [5]. The classical mechanism of ferroptosis involves the glutathione peroxidase 4 (GPX4) regulatory pathway. Studies have shown that inhibiting the function of GPX4 in cells leads to the accumulation of large amounts of lipid peroxides and occurrence of ferroptosis that causes severe and irreversible damage to the cell membranes, leading to cell death [5, 7].

Several studies have reported a close relationship between ferroptosis and microRNAs (miRNAs). miRNAs regulate potential ferroptosis pathways including the expression of mitochondria-related proteins, iron metabolism, glutathione metabolism, and lipid peroxidation [8, 9]. One such member of the miRNA family is miR-139-5p. Abnormal regulation of miR-139-5p has been reported in various malignant tumours, including breast, liver, parathyroid, head and neck, and colorectal cancer [10–12]. However, whether miR-139-5p participates in the regulation of cellular ferroptosis and interferes with glioma development via ferroptosis has rarely been reported.

This study explored the relationship between miR-139-5p and ferroptosis in glioma cells as well as the specific mechanism by which miR-139-5p regulates ferroptosis via downregulating 3-hydroxy-3-methyl-glutaryl-coenzyme A (HMG-CoA) reductase (HMGCR). This study also revealed the synergistic effect of miR-139-5p and simvastatin in the treatment of glioma through ferroptosis. These findings provide new targets and strategies for the clinical treatment of glioma.

## Results

### Gliomas with low miR-139-5p expression predict a worse prognosis

**Fig. 1:**
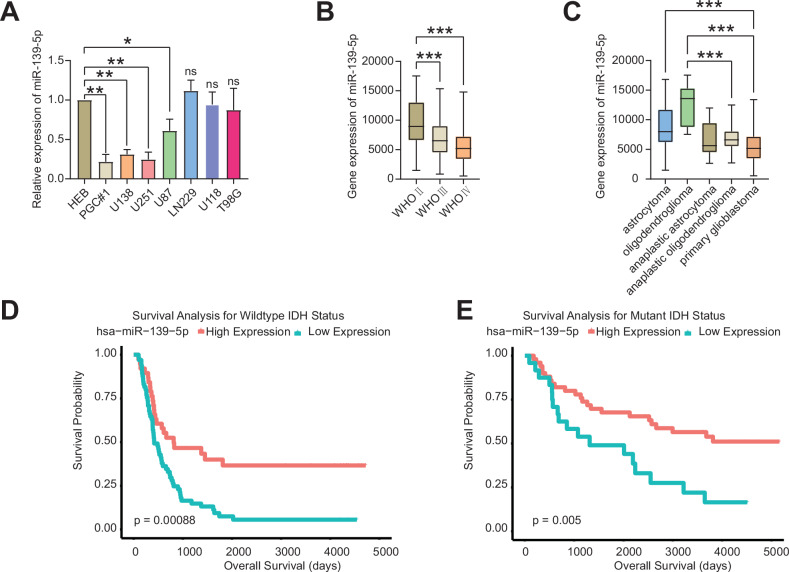
Gliomas with low miR-139-5p expression predict a worse prognosis. **A** qRT-PCR analysis demonstrated the expression levels of miR-139-5p across various glioma cell lines. Results are normalized to U6. **B**, **C** The CGGA database shows that the expression of miR-139-5p is different in various grades or types of glioma cells. **D**, **E** Survival analysis of the CGGA database indicated that the lower the expression level of miR-139-5p, the worse the prognosis of the patients with either wild-type IDH status or mutant IDH status. Data are presented as mean ± standard deviation (SD) from three independent experiments. ***p < 0.001, **p < 0.01, *p < 0.05.

To investigate the differences in miR-139-5p expression levels among several glioma cell lines and normal human brain glial (HEB) cells, we first measured the gene expression levels of each cell type using qRT-PCR (Fig. 1A). The results indicated that the miR-139-5p expression levels in U251, U138 and PGC#1 cells were lowest among HEB cells and other glioma cells. Representative MRI features, gross morphological characteristics, and corresponding hematoxylin-eosin (H&E)-stained histopathological sections of PGC#1-derived tumour specimens were documented in Fig. S1A–C. The morphological characteristics of PGC#1 cells were presented in Fig. S1,D. However, the expression level of miR-139-5p in LN229 cells was not significantly different from that of HEB. We analysed miR-139-5p expression in gliomas of different WHO grades and tissue types using the Chinese Glioma Genome Atlas (CGGA) database. The results showed that miR-139-5p expression levels were significantly lower in WHO III and WHO IV gliomas than in WHO II gliomas. Furthermore, miR-139-5p expression levels were significantly lower in anaplastic oligodendrogliomas and primary glioblastomas with a higher degree of malignancy than in astrocytomas and oligodendrogliomas with a lower degree of malignancy (Fig. 1B, C). Survival analysis using the CGGA database indicated that patients with low miR-139-5p expression had a worse prognosis than those with high expression in gliomas with wild-type IDH status or mutant IDH status (Fig. 1D, E), and in both primary and recurrent gliomas (Fig. S1E). Thus, gliomas with a higher malignancy or grade exhibit a significantly lower expression level of miR-139-5p, which predicts a worse prognosis.

### Overexpression of miR-139-5p promotes ferroptosis in glioma cells

**Fig. 2:**
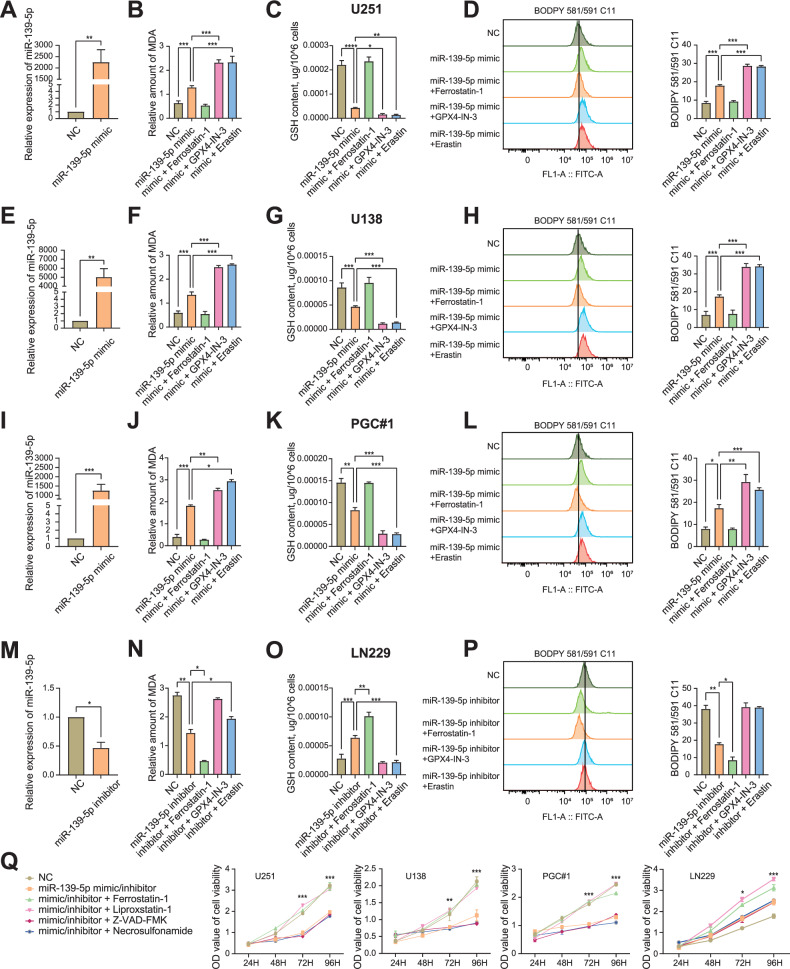
Overexpression of miR-139-5p promotes ferroptosis in glioma cells. **A**, **E**, **I**, **M**, **Q** qRT-PCR showed that 50 nM miR-139-5p mimic was transfected into U251, U138 and PGC#1 cells and increased the expression of miR-139-5p. On the contrary, miR-139-5p inhibitor decreased the expression of miR-139-5p in LN229 cells. Results are normalized to U6 and GAPDH. **B**, **F**, **J**, **N** The MDA content in U251, U138, PGC#1 and LN229 cells was detected using an MDA kit. **C**, **G**, **K**, **O** The GSH content in U251, U138, PGC#1 and LN229 cells was detected using a GSH kit. **D**, **H**, **L**, **P** The Flow cytometry histogram showed the lipid peroxidation level of U251, U138, PGC#1 and LN229 cells. **Q** CCK-8 assay revealed the proliferative activity of U251, U138, PGC#1 and LN229 cells under different treatments. Data are presented as mean ± standard deviation (SD) from three independent experiments. ***p < 0.001, **p < 0.01, *p < 0.05.

In order to increase or decrease the expression of miR-139-5p in glioma cells, U251, U138, PGC#1 and LN229 cells were transfected with 50 nM miR-139-5p mimic or inhibitor for 72 h, and the expression of miR-139-5p was detected by qRT-PCR. The results indicated that the expression of miR-139-5p significantly increased in U251, U138 and PGC#1 cells after miR-139-5p mimic treatment compared to the control group, but the expression of miR-139-5p in LN229 cells was significantly decreased after treatment with miR-139-5p inhibitor (Fig. 2A, E, I, M). Malondialdehyde (MDA) is a metabolite of oxidised lipids and is commonly used as an indicator of lipid peroxidation and ferroptosis [13]. Glutathione (GSH) is a crucial antioxidant in cells that plays a role in various physiological processes. Its content can be measured to assess the progression of ferroptosis [14]. To detect changes in MDA and GSH content in glioma cells, we used relevant assay kits. The results showed that miR-139-5p overexpression significantly increased the MDA content in U251, U138 and PGC#1 cells, while significantly decreasing the GSH content compared to that in the control group. On the contrary, the decreased expression of miR-139-5p in LN229 cells inhibited the production of MDA and promoted the production of GSH (Fig. 2B, C, F, G, J, K, N, O). These results indicated that miR-139-5p overexpression upregulated the content of oxidized lipids and reduced the amount of reducing molecules in glioma cells, but reducing miR-139-5p expression had the opposite effect. Excessive accumulation of lipid peroxides exceeding the cellular detoxification capacity leads to membrane damage and subsequent ferroptosis [15]. BODIPY 581/591 C11 kit and flow cytometry were used to detect intracellular lipid peroxidation. The results showed that overexpression of miR-139-5p increased lipid peroxidation in U251, U138 and PGC#1 cells, and decreased expression of miR-139-5p inhibited lipid peroxidation in LN229 cells (Fig. 2D, H, L, P). Reactive oxygen species (ROS) are peroxides that contain or easily form oxygen free radicals. Under the stimulation of harmful factors, the intracellular ROS level will rise sharply, and then cause cell damage and death [16, 17]. The intracellular ROS levels were measured using flow cytometry. The results indicated that the miR-139-5p mimic group had significantly higher ROS levels than the control group in the U251 and U138 cells (Fig. S2A, B). To observe the difference of mitochondrial morphology in U251 and U138 cells after miR-139-5p overexpression, TEM was used to clearly show the appearance of mitochondria in U251 and U138 cells (Fig. S2C). The results showed that cells in miR-139-5p mimic group had significant smaller mitochondria and higher mitochondrial membrane density, reduced or disappeared mitochondrial cristae, and even mitochondrial outer membrane rupture. The above results were suggested that miR-139-5p was involved in the activation of ferroptosis in gliomas. In addition, Ferrostatin-1, a ferroptosis inhibitor, rescued the ferroptosis caused by miR-139-5p overexpression in U251, U138 and PGC#1 cells. And miR-139-5p mimic had a synergistic effect on the activation of ferroptosis with ferroptosis inducers (FINs) such as GPX4-IN-3 and Erastin. In LN229 cells, miR-139-5p inhibitor synergistically inhibited ferroptosis with Ferrostatin-1, and FINs rescued the ferroptosis attenuation caused by miR-139-5p inhibitor (Fig. 2B–P). Furthermore, ferroptosis resulted in a marked decrease in cell proliferative activity. The results of Cell Counting Kit-8 (CCK-8) assay showed that the proliferative activity of U251, U138 and PGC#1 cells in the miR-139-5p mimic group was significantly lower than that in the control group after 48 h. At the same time, ferroptosis inhibitors such as Ferrostatin-1 and Liproxstatin-1 rescued the decline in cell viability, but apoptosis inhibitor Z-VAD-FMK and necrosis inhibitor Necrosulfonamide did not. Downregulation of miR-139-5p expression in LN229 cells increased cell proliferation. Treatment with miR-139-5p inhibitor and ferroptosis inhibitors further enhanced cell proliferation, but apoptosis inhibitor and necrosis inhibitor had no significant effect on the proliferative activity (Fig. 2Q). TUNEL assay was used to stain apoptotic cells for further detection of apoptosis levels in glioma cells. The results showed that the apoptosis level of glioma cells was not significantly different whether the expression of miR-139-5p was increased or decreased (Fig. S2D). Taken together, these evidences demonstrated that miR-139-5p overexpression solely activated ferroptosis in glioma cells rather than apoptosis or necrosis.

### miR-139-5p inhibits cholesterol synthesis in glioma cells by decreasing the expression of HMGCR

**Fig. 3:**
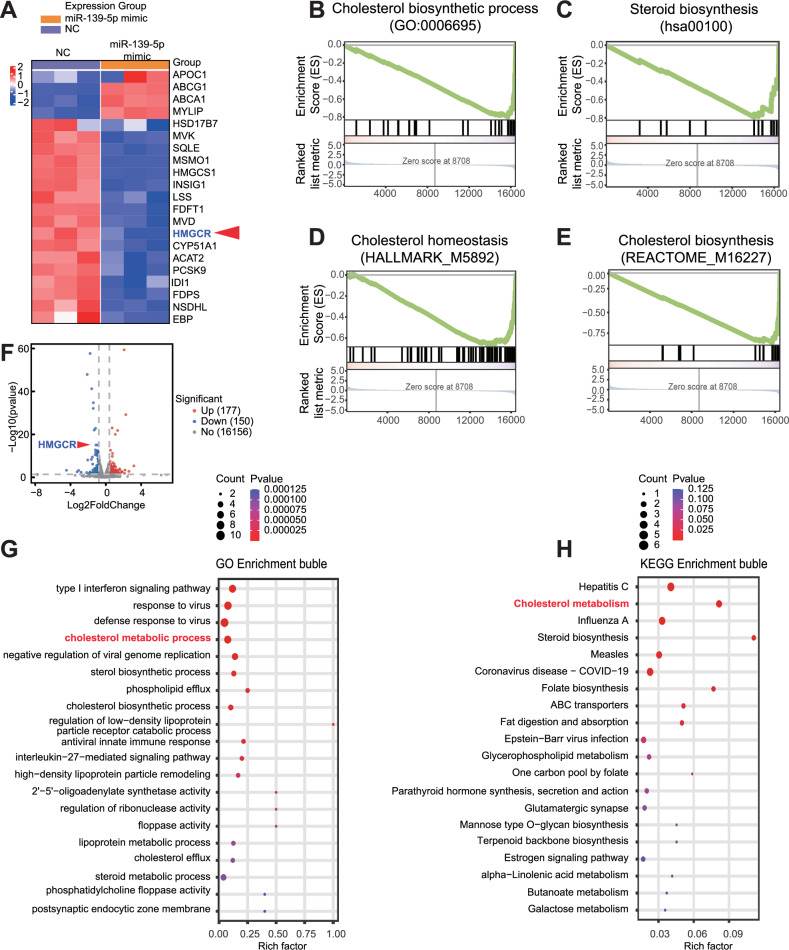
miR-139-5p inhibits cholesterol synthesis in glioma cells by decreasing the expression of HMGCR. **A** Differential gene cluster heat maps in transcriptome sequencing showed downregulated expression of genes related to cholesterol biosynthesis such as HMGCR in miR-139-5p overexpressed glioma cells. **B**–**E** GSEA gene set enrichment analysis revealed that genes after miR-139-5p mimic treatment were mostly enriched in gene sets downregulating cholesterol biosynthesis and other pathways. **F** The differential gene volcano map indicates that the expression of HMGCR in miR-139-5p overexpressed cells was downregulated. **G**, **H** Differential gene GO enrichment bubble map and differential gene KEGG enrichment bubble map showed that miR-139-5p overexpressing cells were enriched in the cholesterol metabolism pathway. Data are presented as mean ± standard deviation (SD) from three independent experiments. ***p < 0.001, **p < 0.01, *p < 0.05.

To investigate how miR-139-5p regulates ferroptosis, we sequenced and analysed all the mRNAs in U251 cells after miR-139-5p overexpression. The heat map of differential gene clustering and the differential gene volcano map showed significant downregulation of key genes involved in cholesterol synthesis, such as HMGCR in miR-139-5p overexpressing cells compared to that in the control group (Fig. 3A, F). GSEA gene set enrichment analysis showed that genes were primarily enriched in gene sets that inhibited cholesterol biosynthesis, steroid biosynthesis, cholesterol homeostasis, and other pathways after miR-139-5p overexpression (Fig. 3B–E). The bubble maps show the differential gene enrichment for Gene Ontology (GO) and Kyoto Encyclopaedia of Genes and Genomes (KEGG), respectively (Fig. 3G, H). These results indicate that overexpression of miR-139-5p leads to the enrichment of differentially expressed genes in the cholesterol metabolism pathway, which is consistent with previous findings. These results suggest that miR-139-5p may indirectly enhance ferroptosis by inhibiting pathways that regulate cellular cholesterol synthesis and HMGCR may be the important downstream molecules. Steroid metabolomics analysis was used to investigate the metabolic changes of glioma cells caused by miR-139-5p overexpression. The results showed that the contents of intracellular steroids such as cholesterol were reduced after treatment with miR-139-5p mimic (Fig. S3A–C). The result of the ferrous ion detection kit indicated that there was no significant difference in ferrous ion levels between the miR-139-5p OE group and the NC group (Fig. S3F). Collectively, these findings suggest that miR-139-5p may modulate cellular ferroptosis through regulation of cholesterol-related metabolic pathways, rather than via direct interference with iron homeostasis mechanisms.

### miR-139-5p promotes ferroptosis by inhibiting the HMGCR expression

**Fig. 4:**
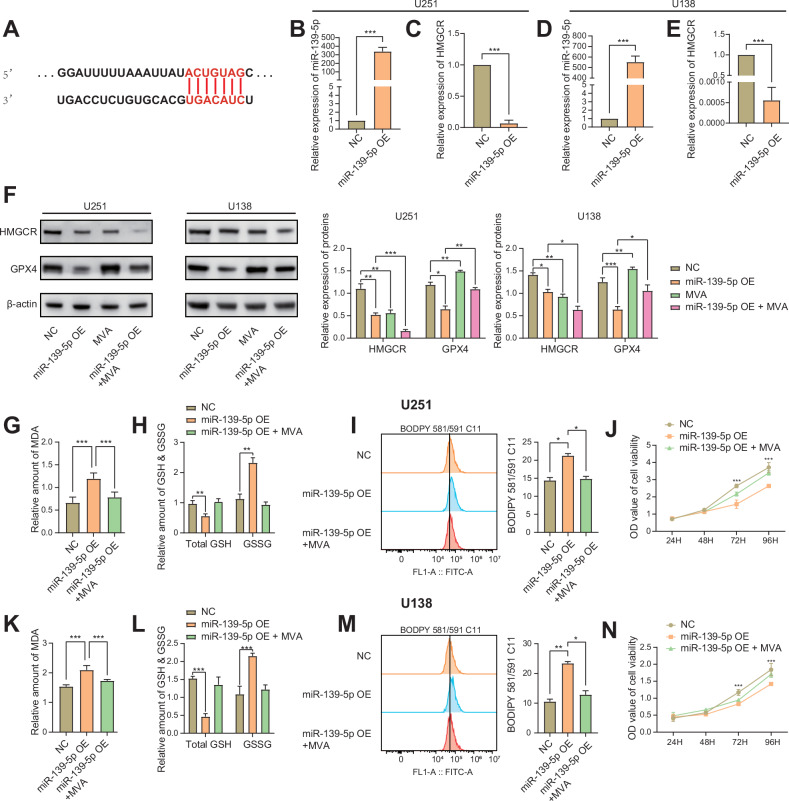
miR-139-5p promotes ferroptosis by inhibiting the HMGCR expression. **A** The binding fragments of miR-139-5p and HMGCR gene were predicted by the TargetScanHuman database. **B**–**E** qRT-PCR assay revealed that the expression of miR-139-5p in U251 and U138 cells transfected with miR-139-5p overexpressed lentivirus was upregulated and the expression of HMGCR was downregulated compared to that in the control group. **F** U251 and U138 cells were treated with 100 μM MVA for 48 h, and the expression levels of related proteins were detected by western blotting. Results of western blotting of U251 and U138 cells were statistically analysed. **G**, **H**, **K**, **L** The relevant kit was used to detect MDA, GSH & GSSG levels in the U251 and U138 cells. **I**, **M** Flow cytometry was used to detect the lipid peroxidation in U251 and U138 cells. Statistical results of lipid peroxidation level in U251 and U138 cells were analysed. **J**, **N** Proliferative activity of U251 and U138 cells was analysed using the CCK-8 assay. Data are presented as mean ± standard deviation (SD) from three independent experiments. ***p < 0.001, **p < 0.01, *p < 0.05.

The TargetScanHuman database revealed the precise binding sites between miR-139-5p and HMGCR mRNA (Fig. 4A). Lentivirus or miR-139-5p inhibitor was transfected into U251, U138, PGC#1 and LN229 cells to upregulate or downregulate miR-139-5p expression. Gene expression levels were determined by qRT-PCR. qRT-PCR results indicated a significant increase in miR-139-5p expression in the miR-139-5p overexpression (OE) group by hundreds of times compared to that in the control group in U251 and U138 cells (Fig. 4B, D). Conversely, HMGCR transcription level in the miR-139-5p OE group of U251, U138 and PGC#1 cells was significantly lower than that in the control group. HMGCR transcription level of LN229 cells of miR-139-5p inhibitor group was significantly higher than that of control group (Figs. 4C, E and S4A). Our qRT-PCR results also showed that miR-139-5p did not affect the mRNA levels of GPX4 or SLC7A11 (Figs. S3F and S4A). U251, U138, PGC#1 and LN229 cells were treated with 100 μM mevalonic acid (MVA), a downstream product of HMGCR, for 48 h for recovery experiments. The expression of proteins was detected using western blotting. The results indicated that HMGCR expression in the miR-139-5p OE group was significantly lower than that in the negative control (NC) group of U251, U138 and PGC#1 cells. miR-139-5p inhibitor increased HMGCR expression in LN229 cells. This suggests that the overexpression of miR-139-5p inhibits HMGCR expression. However, when MVA was used alone, it led to a decrease in the expression of HMGCR in the above cell lines. Additionally, the expression level of HMGCR was lower in the miR-139-5p OE and 100 μM MVA co-treated (miR-139-5p OE + MVA) group than in the miR-139-5p OE group of U251, U138 and PGC#1 cells. The combination of miR-139-5p inhibitor and MVA decreased the expression of HMGCR in LN229 cells compared with miR-139-5p inhibitor group. These findings demonstrate that miR-139-5p overexpression significantly decreases the expression of HMGCR, while MVA treatment additionally suppresses HMGCR expression through negative feedback regulatory mechanisms (Figs. 4F and S4B). Furthermore, the expression level of GPX4 was significantly lower in U251, U138 and PGC#1 cells in the miR-139-5p OE group than in the NC group. And miR-139-5p inhibitor increased GPX4 expression in LN229 cells. This indicates that the overexpression of miR-139-5p inhibits GPX4 protein expression and promoted ferroptosis. MVA alone treatment significantly elevated GPX4 expression levels in the aforementioned cell lines. GPX4 expression was higher in the miR-139-5p OE + MVA group than in the miR-139-5p OE group of U251, U138 and PGC#1 cells. miR-139-5p inhibitor and MVA further increased GPX4 expression in LN229 cells. This suggests that MVA reverses the ferroptosis activated by miR-139-5p overexpression (Fig. 4F) (Fig. S4B). The MDA assay results indicated that the MDA content of the miR-139-5p OE group was significantly higher than the NC group in the U251, U138 and PGC#1 cells. MDA content of LN229 cells of miR-139-5p inhibitor group was significantly lower than that of control group. Furthermore, MDA content in the miR-139-5p OE + MVA group was not significantly different from that in the NC group of U251, U138 and PGC#1 cells. miR-139-5p inhibitor and MVA further reduced the MDA content of LN229 cells (Figs. 4G, K and S4C, G). In U251, U138 and PGC#1 cells, the GSH and GSSG assay showed:(a) significant decrease in GSH content, (b) significant increase in GSSG content in the miR-139-5p OE group compared to the NC group. miR-139-5p inhibitor increased the GSH content and decreased the GSSG content of LN229 cells. Moreover, in U251, U138 and PGC#1 cells, the GSH and GSSG assay showed:(a) significant increase in GSH content, (b) significant decrease in GSSG content in the miR-139-5p OE + MVA group compared to the miR-139-5p OE group. miR-139-5p inhibitor and MVA further increased the GSH content and further decreased the GSSG content of LN229 cells (Figs. 4H, L and S4D, H). BODIPY 581/591 C11 kit and flow cytometry were used to detect intracellular lipid peroxidation. The results suggested that miR-139-5p overexpression promoted lipid peroxidation in U251, U138 and PGC#1 cells, while MVA reversed the increase of lipid peroxidation. miR-139-5p inhibitor inhibited lipid peroxidation in LN229 cells, and MVA further downregulated lipid peroxidation levels (Figs. 4I, M and S4E, I). In addition, ROS levels in U251 and U138 cells in each group were detected by flow cytometry. The results showed that ROS levels in the miR-139-5p OE group in U251 and U138 cells were significantly higher than those in the NC group. The ROS levels in the miR-139-5p OE + MVA group were significantly lower than those in the miR-139-5p OE group (Fig. S5A, B). The CCK-8 assay results indicated that the proliferation rate of U251, U138 and PGC#1 cells in the miR-139-5p OE group was significantly lower than that in the NC and miR-139-5p OE + MVA group after 72 h. The proliferative activity of LN229 cells in miR-139-5p inhibitor+MVA group was significantly higher than that in the control group after 72 h. (Figs. 4J, N and S4F, J). These results indicated that miR-139-5p overexpression inhibited the expression of HMGCR, GPX4 and promoted glioma cells ferroptosis, which was reversed by MVA.

### miR-139-5p synergistically promotes ferroptosis and inhibits cholesterol synthesis with simvastatin

**Fig. 5:**
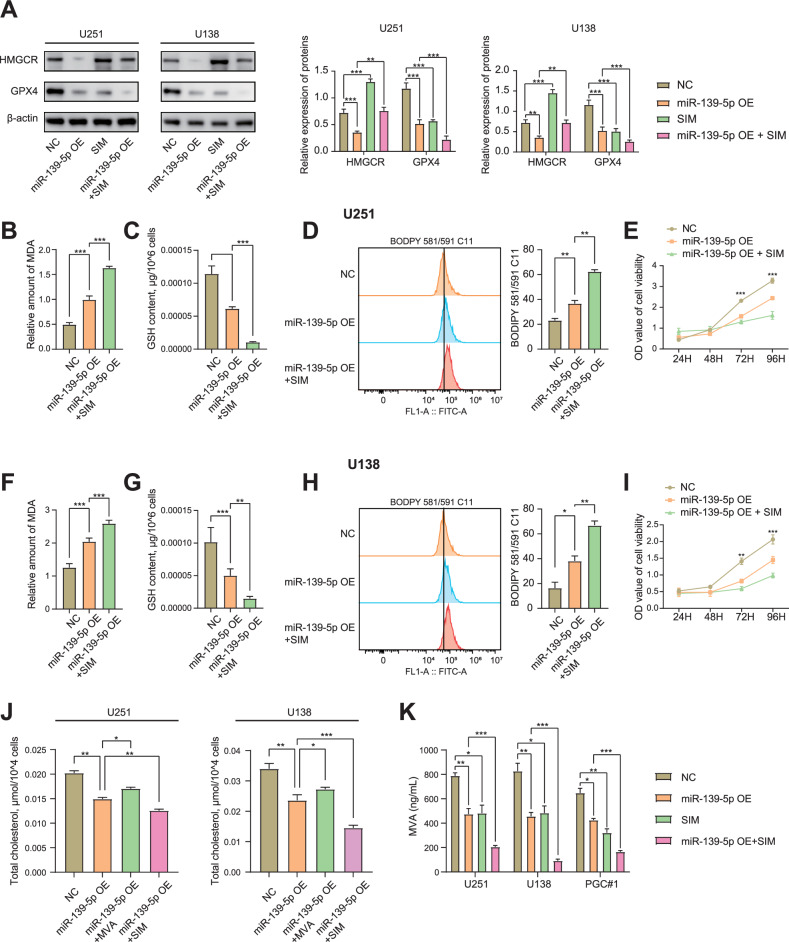
miR-139-5p synergistically promotes ferroptosis and inhibits cholesterol synthesis with simvastatin. **A** U251 and U138 cells were treated with 10 μM SIM for 48 h, and the expression levels of related proteins were detected by western blotting. Results of western blotting of U251 and U138 cells were statistically analysed. **B**, **C**, **F**, **G** The relevant kit was used to detect MDA, GSH levels in the U251 and U138 cells. **D**, **H** Flow cytometry was used to detect the lipid peroxidation levels in U251 and U138 cells. Statistical results of lipid peroxidation detection in U251 and U138 cells. **E**, **I** Proliferative activity of U251 and U138 cells was analysed using the CCK-8 assay. **J** The relevant kit was used to detect cholesterol levels in the U251 and U138 cells. **K** LC–MS/MS was employed to quantitatively analyze the intracellular levels of mevalonic acid (MVA). Data are presented as mean ± standard deviation (SD) from three independent experiments. ***p < 0.001, **p < 0.01, *p < 0.05.

U251, U138, PGC#1 and LN229 cells were treated with 10 μM simvastatin (SIM), a classical inhibitor of HMGCR, for 48 h. The expression of proteins was detected using western blotting. The results demonstrated that in U251, U138, and PGC#1 cells, HMGCR expression was significantly downregulated in the miR-139-5p OE group compared to the NC group, while the SIM group showed an opposite trend with markedly elevated HMGCR levels compared to the NC group. And the expression level of HMGCR was higher in the miR-139-5p OE and 10 μM SIM co-treated (miR-139-5p OE + SIM) group than that in the miR-139-5p OE group. In LN229 cells, both miR-139-5p inhibitor and SIM increased the expression of HMGCR, and the combination of miR-139-5p inhibitor and SIM further elevated the level of HMGCR. This indicated that miR-139-5p overexpression downregulated HMGCR expression, whereas SIM inhibition of HMGCR enzymatic activity resulted in negative feedback upregulation of HMGCR (Figs. 5A and S6A). Furthermore, GPX4 expression levels in both the miR-139-5p OE and SIM groups were significantly downregulated compared to the NC group across U251, U138, and PGC#1 cell lines. GPX4 expression was lower in the miR-139-5p OE + SIM group than that in the miR-139-5p OE group. In LN229 cells, compared to the NC group, miR-139-5p inhibitor upregulated the expression of GPX4, while SIM downregulated GPX4 expression. The GPX4 levels in the miR-139-5p inhibitor+SIM group showed no significant difference compared to the NC group. This suggests that miR-139-5p overexpression downregulates GPX4 expression, which is further suppressed by SIM in a synergistic manner with miR-139-5p (Figs. 5A and S6A). The MDA assay results indicated that the MDA content of the miR-139-5p OE group was significantly higher than the NC group in the U251, U138 and PGC#1 cells. Furthermore, MDA content in the miR-139-5p OE + SIM group was higher than that in the miR-139-5p OE group. The MDA content of LN229 cells of miR-139-5p inhibitor group was lower than that of NC group, and the decrease of MDA content was reversed by SIM (Fig. 5B, F). The GSH assay kit results indicated that the GSH content of the miR-139-5p OE group was significantly lower than the NC group in the U251, U138 and PGC#1 cells. Moreover, GSH content in the miR-139-5p OE + SIM group was lower than that in the miR-139-5p OE group. The GSH content of LN229 cells of miR-139-5p inhibitor group was higher than that of NC group, and the increase of GSH content was reversed by SIM (Figs. 5C, G and S6C, G). BODIPY 581/591 C11 kit and flow cytometry were used to detect intracellular lipid peroxidation. The results suggested that miR-139-5p overexpression promoted lipid peroxidation in U251, U138 and PGC#1 cells, while SIM further increased lipid peroxidation. miR-139-5p inhibitor inhibited lipid peroxidation in LN229 cells, and SIM reversed the decrease of lipid peroxidation levels (Figs. 4I, M and S4E, I). In addition, ROS levels in U251 and U138 cells in each group were detected by flow cytometry. The results showed that ROS levels in the miR-139-5p OE group in U251 and U138 cells were significantly higher than those in the NC group. The ROS levels in the miR-139-5p OE + SIM group were significantly higher than those in the miR-139-5p OE group (Fig. S5C, D). The CCK-8 assay results indicated that the proliferation rate of U251, U138 and PGC#1 cells in the miR-139-5p OE group was significantly lower than that in the NC group, and higher than that in the miR-139-5p OE + SIM group after 72 h. The proliferative activity of LN229 cells in miR-139-5p inhibitor group was significantly higher than that in the control group and miR-139-5p inhibitor+SIM group after 96 h. (Figs. 5E, I and S6E, I). These results indicated that miR-139-5p and SIM further promoted ferroptosis synergistically in glioma cells. The results of the total cholesterol assay indicated that the cholesterol content of the miR-139-5p OE group was significantly lower than the NC group in U251, U138 and PGC#1 cells, and the cholesterol content of the miR-139-5p OE + MVA group was higher than the miR-139-5p OE group. The cholesterol content in the miR-139-5p OE + SIM group was significantly lower than that in the NC and miR-139-5p OE groups. The cholesterol content of LN229 cells of miR-139-5p inhibitor group was higher than that of NC group, and MVA further raises cholesterol levels. SIM reversed the increase in cholesterol levels caused by miR-139-5p inhibitor (Figs. 5J and S6J). This indicated that miR-139-5p overexpression inhibited cholesterol synthesis in glioma cells, MVA reversed the inhibition of cholesterol synthesis by miR-139-5p, SIM and miR-139-5p synergistically inhibited cholesterol synthesis further. Liquid chromatography-tandem mass spectrometry (LC–MS/MS) analysis demonstrated that miR-139-5p OE or SIM alone significantly reduced MVA levels in U251, U138 and PGC#1 cells, with synergistic attenuation observed in the miR-139-5p OE + SIM group. Regarding isopentenyl pyrophosphate (IPP), both miR-139-5p OE and SIM alone substantially diminished intracellular IPP content, whereas MVA alone conversely elevated IPP accumulation. Notably, the miR-139-5p OE + MVA group showed no significant difference in IPP levels compared to NC group, while the miR-139-5p OE + SIM caused a more pronounced decrease in IPP content (Figs. 5K and S3D). To evaluate the activity of selenocysteine tRNA (sec-tRNA) in glioma cells under different treatments, this study employed a luciferase reporter system to analyze U251, U138 and PGC#1 cells. The results indicated that both miR-139-5p OE and SIM treatment alone reduced sec-tRNA activity, while MVA treatment alone enhanced sec-tRNA activity. When miR-139-5p OE was combined with MVA, there was no significant difference in sec-tRNA activity compared to the NC group. However, the combination of miR-139-5p OE and SIM further decreased sec-tRNA activity (Fig. S3E). Overall, miR-139-5p and SIM synergistically reduced sec-tRNA activity by inhibiting the MVA pathway, which affected the protein synthesis of GPX4 and ultimately promoted ferroptosis.

### miR-139-5p promotes ferroptosis and inhibits cholesterol biosynthesis and tumour progression in vivo

**Fig. 6:**
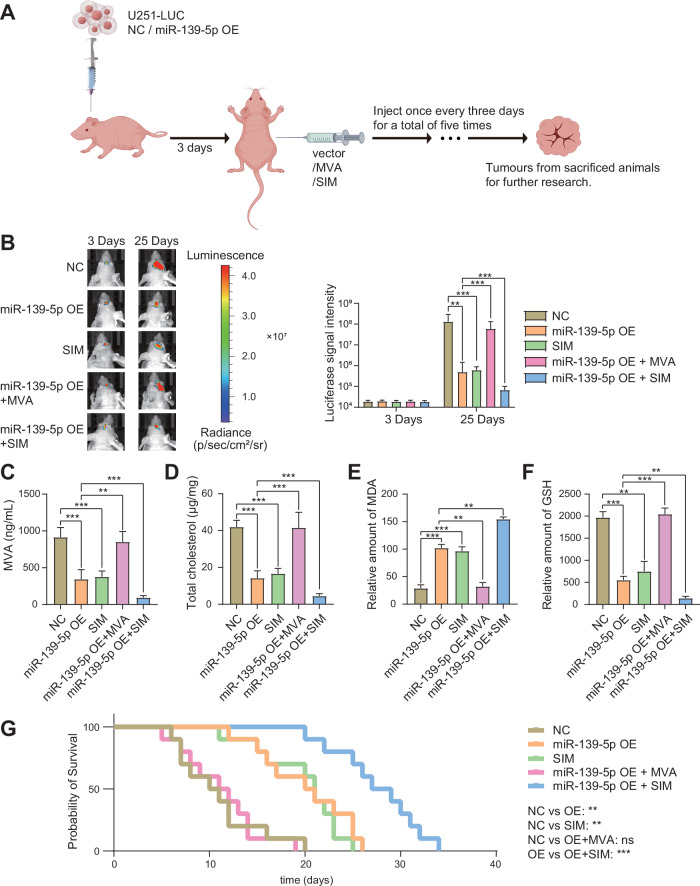
miR-139-5p promotes ferroptosis and inhibits cholesterol biosynthesis and tumour progression in vivo. **A** Schematic diagram of animal experimental procedures. Created with www.figdraw.com. **B** 100 mg/kg MVA or 10 mg/kg SIM were injected intraperitoneally every three days to treat mice in the corresponding group, for five times. Bioluminescence imaging indicated the size of intracranial tumours under different treatments. The luminescent signal intensity of each group of glioma-bearing mice was statistically analysed. **C** LC–MS/MS was employed to quantify the levels of MVA in tumour tissues. **D** The total cholesterol content of the tumour tissues was detected using the cholesterol kit. These results align with in vitro experiments. **E** MDA kit was used to detect the content of MDA in tumour tissues. **F** The GSH content in tumour tissues was detected by GSH kit. **G** The results of the Balb/c nude mouse survival analysis were consistent with those of the in vitro experiments. Data are presented as mean ± standard deviation (SD) from three independent experiments. ***p < 0.001, **p < 0.01, *p < 0.05.

To investigate whether miR-139-5p can hinder glioma progression by regulating ferroptosis in vivo, we subcutaneously injected U251 cells or U251 miR-139-5p OE cells into 20 BALB/c nude mice (3–5 weeks, 20–30 g) and orthotopically injected mCherry-LUC Vector-transfected U251 cells or mCherry-LUC Vector-transfected U251 OE cells into another 50 BALB/c nude mice to establish glioma animal models. The mice were divided into the five groups as NC, miR-139-5p OE, SIM, miR-139-5p+MVA, miR-139-5p+SIM. Mice in the miR-139-5p+MVA group were intraperitoneally injected with 100 mg/kg of MVA every three days, for five injections. The mice in the SIM group and the miR-139-5p OE + SIM group were intraperitoneally injected with 10 mg/kg SIM every three days for five injections (Fig. 6A). The results of bioluminescent imaging suggested that the luminescence intensity of intracranial tumours in miR-139-5p OE group and SIM group was significantly lower than that in NC group after 25 days. There was no significant difference in luminescence intensity between miR-139-5p OE + MVA group and NC group. The intracranial luminescence intensity of miR-139-5p OE + SIM group was the lowest (Fig. 6B). Intracranial tumour tissues were collected by dissection after mice death. After 15 days, another 20 mice were anesthetized with 40–80 mg/kg phenobarbital sodium via intraperitoneal injection and sacrificed, and the subcutaneous tumour tissue was dissected. The tumour tissue weight and volume were significantly lower in the miR-139-5p OE group than in the NC group. In the miR-139-5p OE + MVA group, the weight and volume of tumour tissues were higher than those in the miR-139-5p OE group, but there was no significant difference compared to the NC group. The weight and volume of tumour tissue in the miR-139-5p OE + SIM group were significantly lower than those in the other three groups (Fig. S7A-C). This indicated that miR-139-5p overexpression and SIM alone treatment inhibited tumour growth in vivo, MVA reversed the inhibitory effect of miR-139-5p, and SIM synergistically inhibited tumour growth with miR-139-5p. The LC-MS/MS analysis of MVA content in tumour tissues demonstrated that both miR-139-5p OE and SIM markedly decreased MVA levels. The combination of miR-139-5p OE with MVA showed no significant difference in MVA levels compared to the NC group. Additionally, the combination of miR-139-5p OE and SIM led to a further reduction in MVA levels (Fig. 6C). Furthermore, we detected total cholesterol in the both intracranial and subcutaneous tumour tissues. The results showed that the total cholesterol content of tumours in the miR-139-5p OE group and SIM group was significantly lower than that in the NC group, and there was no significant difference between the NC group and the miR-139-5p OE + MVA group. The total tumour cholesterol content in the miR-139-5p OE + SIM group was significantly lower than that in the other three groups. These results are consistent with those in vitro experiments (Figs. 6D and S7D). The levels of MDA and GSH in intracranial and subcutaneous tumour tissues were subsequently measured to assess ferroptosis. The results showed that MDA content in tumour tissues of miR-139-5p OE group and SIM group was significantly higher than that of NC group. There was no significant difference in MDA content between miR-139-5p OE + MVA group and NC group, and MDA content in miR-139-5p OE + SIM group was significantly higher than that in the other three groups (Figs. 6E and S7E). The results showed that the GSH content in tumour tissues of miR-139-5p OE group and SIM group was significantly lower than that of NC group, and there was no significant difference between miR-139-5p OE + MVA group and NC group. The GSH content of miR-139-5p OE + SIM group was significantly lower than that of the other three groups (Fig. 6F) (Fig. S7F). These results suggested that miR-139-5p overexpression promoted ferroptosis in glioma tissues in vivo, MVA reversed the ferroptosis caused by miR-139-5p overexpression, and SIM synergistically promoted ferroptosis in tumour tissues with miR-139-5p. The results of the survival analysis indicated that the median survival time of mice in the miR-139-5p OE group and SIM group was significantly longer than that of mice in the NC group. Moreover, the median survival time of mice in the miR-139-5p OE + MVA group was not significantly different from that of mice in the NC group. However, the median survival time of the miR-139-5p OE + SIM group was significantly longer than the other three groups (Fig. 6G). These results suggest that miR-139-5p can impede the progression of glioma and prolong the survival of mice by inhibiting HMGCR expression to activate ferroptosis and inhibit cholesterol synthesis in vivo.

## Discussion

Gliomas are the most common and dangerous malignant tumours of the central nervous system. And the median survival of patients with WHO grade IV gliomas is still less than two years [18, 19]. Therefore, it is necessary for researchers to continue to explore the various mechanisms of glioma progression and effective treatment methods. In this study, a postoperative tumour sample from a patient was collected, and primary cells, designated as PGC#1, were extracted from the sample. The MRI imaging, gross appearance, microscopic morphology, and pathological analysis of the tumour sample were consistent with the characteristics of IDH-1 wild-type glioblastoma. In order to explore the effect of miR-139-5p on glioma progression. In this study, we extracted RNA from multiple glioma cells and human normal glial cell HEB, and detected the expression of miR-139-5p in these cells by qRT-PCR. The results showed that the expression of miR-139-5p in PGC#1, U251, U138 and U87 glioma cells was lower than that in HEB, and the expression of miR-139-5p in PGC#1, U251 and U138 cells was the lowest. The expression of miR-139-5p in other cells was not significantly different from that of HEB cells, such as LN229 cells. CGGA database information indicated that the expression level of miR-139-5p decreased with the increase of malignancy degree or WHO grade of glioma. Survival analysis showed that lower miR-139-5p expression in patients with IDH wild-type gliomas or IDH-mutant type gliomas predicted worse prognosis. Wang et al. also found that when the expression of miR-139-5p is increased or its function is enhanced, the growth of gliomas is inhibited both in vivo and in vitro [11, 20]. These results suggest that miR-139-5p may function as a tumour suppressor gene in gliomas. And this study selected U251, U138, PGC#1 and LN229 cells for subsequent studies.

Ferroptosis is a form of non-apoptotic programmed cell death, characterised by the accumulation of lipid peroxidation or iron. If Fe^2+^ is overloaded in the cell, it can produce excess ROS beyond the tolerance limit of the cell through the Fenton reaction [21, 22]. This results in the accumulation of excessive lipid peroxides, particularly the peroxidation of polyunsaturated fatty acids (PUFAs) on the cell membrane, ultimately causing a breakdown of the REDOX balance and cell death [4, 23]. In this study, we investigated the relationship between miR-139-5p and ferroptosis. U251, U138 and PGC#1 cells were treated with miR-139-5p mimic to increase the expression of miR-139-5p in the cells, and LN229 cells were treated with miR-139-5p inhibitor to decrease the expression of miR-139-5p, then qRT-PCR was used to detect the gene expression. The results showed that miR-139-5p mimic or miR-139-5p inhibitor indeed overexpressed or downregulated miR-139-5p in cells. Lipid peroxidation is one of the markers of ferroptosis, and MDA is often considered as an index to detect lipid peroxidation. Therefore, when MDA content increases in cells or tissues, the ferroptosis level also increases [24]. GSH protects cells from toxic substances containing oxygen free radicals such as lipid peroxides under physiological conditions, so GSH often inhibits ferroptosis [25]. After increasing or decreasing the expression level of miR-139-5p in cells, we detected the changes of MDA and GSH contents with corresponding kits, and the difference of lipid peroxidation or ROS in cells was detected by flow cytometry. Overexpression of miR-139-5p in U251, U138 and PGC#1 cells resulted in increased levels of lipid peroxidation, MDA and ROS and decreased levels of GSH, which represented the activation of ferroptosis. Since mitochondria are the main site of ROS production in most cells, the involvement of mitochondria is crucial in the process of ferroptosis [26]. We used TEM to observe the morphological changes of mitochondria in U251 and U138 cells after miR-139-5p overexpression, and found that compared with the NC group, the mitochondria showed significant shrinkage, mitochondrial cristae shrinkage, increased mitochondrial membrane density, and even mitochondrial outer membrane rupture. CCK-8 results also suggested that the proliferation activity of U251, U138 and PGC#1 cells was significantly decreased after miR-139-5p overexpression. In addition, our study also found that miR-139-5p overexpression had a synergistic effect on promoting ferroptosis with a variety of FINs. Moreover, ferroptosis inhibitors reversed the ferroptosis activated by miR-139-5p overexpression, but apoptosis inhibitors or necrosis inhibitors did not. The effect of reducing the expression of miR-139-5p in LN229 cells was opposite to that of the previous three cells. TUNEL staining also indicated that miR-139-5p did not affect the apoptosis of glioma cells. Therefore, we can conclude that miR-139-5p can inhibit tumour progression by solely activating ferroptosis in glioma cells. And the specific mechanism of miR-139-5p regulating ferroptosis needs to be further explored.

To clarify the mechanism by which miR-139-5p promotes ferroptosis in glioma cells, transcriptome sequencing was performed after miR-139-5p overexpression in U251 cells. The results showed that the expression levels of key genes associated with cholesterol synthesis, such as HMGCR, were significantly lower in U251 cells overexpressing miR-139-5p than in the control group. The differentially expressed genes were mainly enriched in gene sets that suppressed cholesterol biosynthesis and other pathways. These results suggested that miR-139-5p may decrease cellular cholesterol production by inhibiting HMGCR expression. Steroid metabolomics analysis also confirmed that miR-139-5p overexpression reduced cellular cholesterol synthesis. In addition, the relationship between intracellular cholesterol and ferroptosis has been extensively studied in recent years and the existing research results suggest that cholesterol can inhibit the occurrence of ferroptosis [27]. Li et al. ‘s findings suggest that 7-dehydrocholesterol, as an intermediate in distal cholesterol synthesis, is a strong inhibitor of ferroptosis and can determine cell sensitivity to ferroptosis [28]. Moreover the mevalonate pathway that is mainly responsible for the synthesis of cholesterol has been considered in the participation of regulation of ferroptosis because isopentenyl pyrophosphate (IPP) as the intermediate products in this pathway is not only a key molecule involved in GPX4 synthesis but also a precursor to the production of the lipid hydroperoxide inhibitor CoQ10 [29]. It is worth noting that HMGCR is a key rate-limiting enzyme in the mevalonate pathway, whose main function is to catalyze the conversion of HMG-CoA to mevalonic acid (MVA) in the biosynthesis of cholesterol [30]. Wang’s team’s findings indicate that BRCC36 inhibits ferroptosis in hepatocellular cancer cells by deubiquitinating HMGCR [31]. On the other hand, the detection of ferrous ions indicated that miR-139-5p had no significant effect on iron metabolism in glioma cells. Therefore, this study suggests that miR-139-5p regulates the MVA pathway by intervening in the expression of HMGCR, indirectly affecting the sensitivity of tumour cells to ferroptosis, rather than through mechanisms related to iron metabolism regulation. However, further studies are required to determine the underlying mechanisms.

The TargetScanHuman database indicated that miR-139-5p and HMGCR mRNA contain gene-binding fragments, which suggests that HMGCR is one of the direct downstream molecules of miR-139-5p. In this study, miR-139-5p expression was stably increased or decreased after transfection of miR-139-5p overexpressing lentivirus or miR-139-5p inhibitor into glioma cells compared to the NC group. Simultaneously, qRT-PCR results indicated that the transcriptional level of HMGCR decreased with the upregulation of miR-139-5p expression. However, the expression level of miR-139-5p had no significant impact on the mRNA levels of GPX4 and SLC7A11. Western blotting results indicated that the overexpression of miR-139-5p reduced HMGCR levels in U251, U138 and PGC#1 cells compared to the NC group. Furthermore, the protein levels of GPX4 in the three cell types in the miR-139-5p OE group were significantly lower than those in the NC group. MVA is a downstream product of HMGCR. Treatment with 100 μM MVA in U251, U138 and PGC#1 cells resulted in the downregulation of HMGCR expression due to the negative feedback regulation, while the expression of GPX4 increased as a consequence of the promoted protein synthesis by MVA. The combination of miR-139-5p OE and MVA further suppressed the expression of HMGCR and also rescued the downregulation of GPX4 induced by miR-139-5p OE alone. The results of MDA, GSH, lipid peroxidation, ROS and CCK-8 assays in this study showed that miR-139-5p overexpression promoted ferroptosis in glioma cells, and MVA reversed the ferroptosis induced by miR-139-5p overexpression. Notably, miR-139-5p OE significantly reduced GSH levels while markedly increasing intracellular GSSG levels compared to the NC group. This further demonstrates that miR-139-5p indirectly suppresses GPX4 expression by downregulating HMGCR, ultimately inducing ferroptosis, rather than through SLC7A11 [32, 33]. GPX4, as a key enzyme specifically responsible for clearing lipid hydroperoxides (LPO), exhibited upregulated expression induced by MVA, which directly reduced the accumulation of ROS such as intracellular peroxidized lipids. Secondary products such as 4-HNE generated through the decomposition of LPO were consequently reduced, leading to the interruption of the cascade amplification reaction of ROS. Ultimately, MVA significantly decreased the total intracellular ROS levels [34, 35]. Downregulating the expression of miR-139-5p in LN229 cells caused the opposite effect. GPX4 acts as a GSH-dependent lipid repair enzyme that neutralizes lipid hydroperoxides, while the cystine-glutamate antiporter (System Xc⁻) provides cysteine precursor for GSH synthesis via cystine uptake. This process prevents the conversion of iron-dependent lipid peroxides into highly active lipid radicals, thereby inhibiting the development of ferroptosis [36–38]. GPX4 is a selenocysteine protein with a selenocysteine active centre. Normal protein function requires selenocysteine participation [39]. Selenocysteine tRNA (Sec-tRNA) plays a key role in the transport of selenocysteine to GPX4. The maturation of Sec-tRNA depends on IPP, an intermediate metabolite of the mevalonate pathway in cholesterol synthesis [39]. This process of maturation requires the specific binding of IPP to the adenine sites of Sec-tRNA precursors. Thereby activation of the mevalonate pathway induced by MVA increased the intracellular IPP content and up-regulated GPX4 expression. This suggests that miR-139-5p induced ferroptosis by reducing HMGCR expression and it is reversed by MVA.

Simvastatin (SIM) is an effective inhibitor of HMGCR and a commonly used cholesterol-lowering drug in clinical practice [30]. In addition, a large number of studies have reported that SIM plays a huge role in inducing ferroptosis in a variety of tumours and hindering the progression of tumours [30, 40]. Kitsugi’s findings indicate that SIM can inhibit the activation of hepatic stellate cells (HSCs) by increasing their sensitivity to ferroptosis [41]. Since miR-139-5p and SIM share a common target molecule HMGCR, this study aims to explore whether miR-139-5p and SIM can synergistically promote ferroptosis in glioma cells. The results of western blotting in this study indicated that miR-139-5p overexpression inhibited HMGCR and GPX4 expression in U251, U138 and PGC#1 cells. SIM upregulated HMGCR expression in cells while simultaneously downregulating GPX4 expression. Moreover, the expression of HMGCR in miR-139-5p OE + SIM group was higher than that in miR-139-5p OE group, while the expression of GPX4 was lower than that in miR-139-5p OE group. Because SIM reduced HMGCR bioactivity through competitive inhibition, and the negative feedback regulatory mechanism of cells upregulated HMGCR expression relatively [42]. On the other hand, miR-139-5p and SIM synergistically inhibited the GPX4 expression. In this study, the related indicators of ferroptosis such as MDA, GSH, lipid peroxidation, ROS and CCK-8 were detected, and the results suggested that miR-139-5p overexpression promoted ferroptosis in U251, U138 and PGC#1 cells, and the combined use of SIM and miR-139-5p further promoted ferroptosis in glioma cells. The results of total cholesterol assay also showed that miR-139-5p and SIM had a synergistic inhibitory effect on cholesterol synthesis. Downregulating the expression of miR-139-5p in LN229 cells caused the opposite effect. The detection results of intracellular MVA, IPP levels and sec-tRNA activity further revealed that both miR-139-5p OE and SIM could reduce the content of MVA and IPP and the activity of sec-tRNA. In contrast, MVA treatment increased the levels of IPP and the activity of sec-tRNA. The combination of miR-139-5p OE and SIM further decreased the content of MVA and IPP and the activity of sec-tRNA. Taken together, this study demonstrated that miR-139-5p regulates the MVA/IPP/sec-tRNA pathway by directly inhibiting HMGCR expression, ultimately reducing GPX4 protein synthesis and promoting cellular ferroptosis. Furthermore, SIM and miR-139-5p exhibited a synergistic effect in promoting cellular ferroptosis, providing novel therapeutic strategies and targets for the clinical treatment of glioma.

The conclusions of the in vivo experiments were consistent with those of the in vitro experiments. The tumour luminescence intensity, volume and weight of BALB/c nude mice in miR-139-5p OE group and SIM group were significantly lower than those in the NC group. 100 mg/kg MVA reversed the inhibitory effect of miR-139-5p on tumour growth. 10 mg/kg SIM and miR-139-5p had a synergistic inhibitory effect on tumour growth. The assays of MDA, GSH, MVA and total cholesterol in tumour tissues and the survival analysis of mice suggested that miR-139-5p overexpression promoted ferroptosis and reduced cholesterol content in tumour tissues. MVA reversed the effects of miR-139-5p. SIM and miR-139-5p have a synergistic effect on promoting tumour ferroptosis and inhibiting cholesterol synthesis. Therefore, miR-139-5p could intervene in the MVA pathway and cholesterol synthesis by downregulating HMGCR expression in vivo, ultimately promoting ferroptosis. SIM and miR-139-5p can synergistically promote ferroptosis and inhibit cholesterol synthesis in glioma, and jointly participate in the treatment of glioma.

### Limitations

The molecules involved in the process of miR-139-5p reducing HMGCR expression in glioma cells and the specific molecular mechanisms remain to be explored. It is worth noting that this study primarily focuses on the regulatory mechanisms of ferroptosis in IDH1 wild-type glioblastomas. Although survival analysis suggests that low expression of miR-139-5p also has predictive value for the prognosis of patients with IDH mutant gliomas, it is important to emphasize that IDH1 mutant tumours may have unique metabolic characteristics. Therefore, the specific mechanism of action of miR-139-5p in IDH mutant gliomas may exhibit heterogeneity, which requires further research through the establishment of IDH1 mutant models for specialized investigation.

## Conclusion

**Fig. 7:**
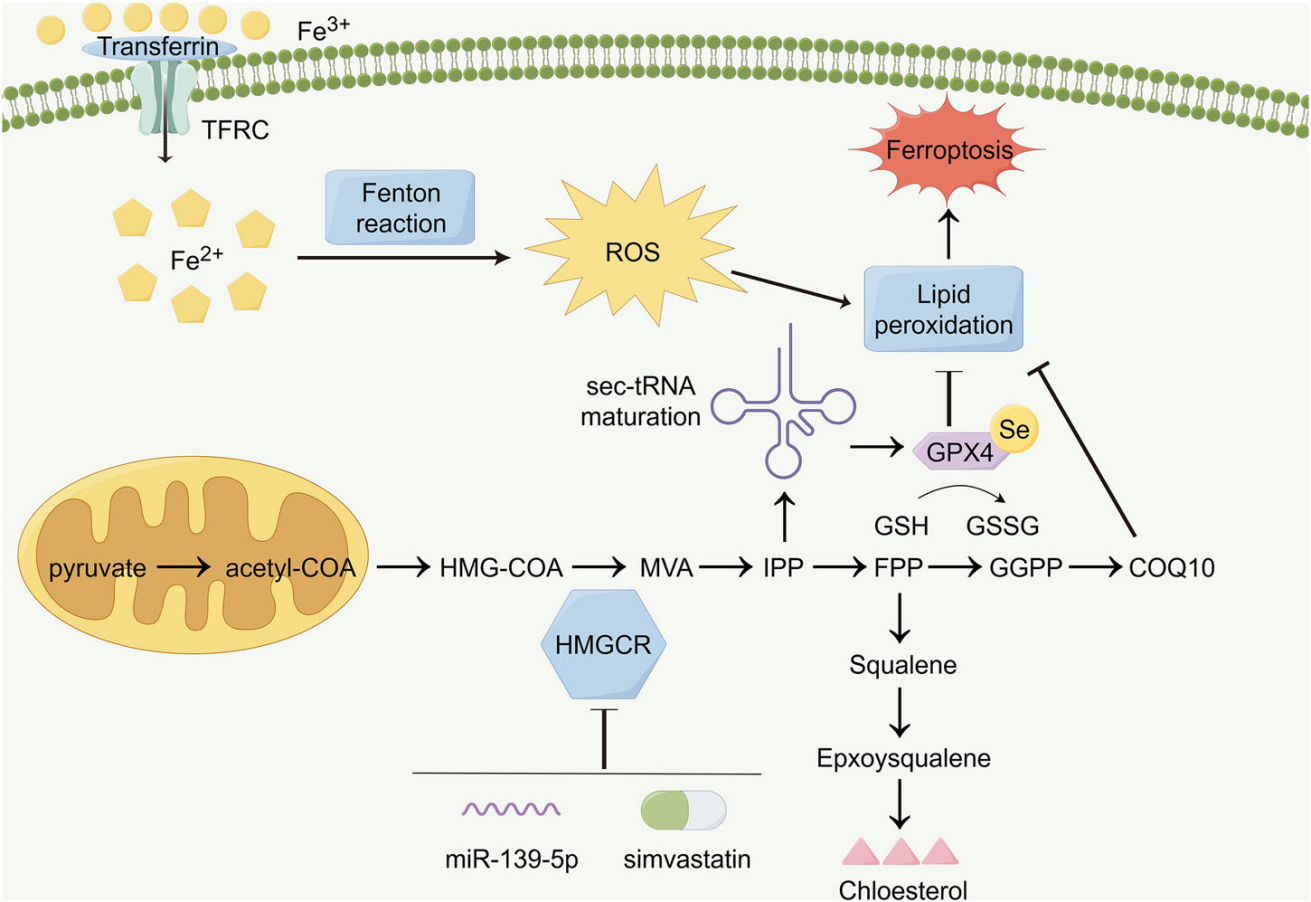
Related schematic diagram of this study. The schematic diagram briefly describes the process of cholesterol synthesis and the specific molecular mechanism by which this pathway regulates the progression of ferroptosis. In addition, miR-139-5p and SIM synergistically inhibited the enzymatic activity of HMGCR to participate in the regulation of cholesterol biosynthesis and cell ferroptosis. Created with www.figdraw.com.

miR-139-5p reduces HMGCR expression in glioma cells and downregulates GPX4 expression. It then promotes the activation of ferroptosis and inhibits cholesterol synthesis in vivo and in vitro, ultimately hindering glioma development. Simultaneously, miR-139-5p can synergistically activate ferroptosis in gliomas with SIM, providing a new therapeutic strategy and target for the clinical treatment of gliomas (Fig. 7).

## Materials and methods

### Cell culture

The glioma cell lines U251, U138 and LN229, purchased from the Chinese Academy of Sciences, were cultured in Dulbecco’s modified Eagle’s medium (DMEM; Gibco, Carlsbad, CA, USA) supplemented with 10% foetal bovine serum (Gibco). Cells were cultured in a modified incubator at a constant temperature of 37 °C and 5% CO_2_. In order to culture primary glioma cells (PGC#1), glioblastoma tissues from post-operative patients in the department of neuro-oncological surgery at Zhujiang Hospital were digested by Accutase (Sigma) at 37 °C for half an hour, and then primary glioma cells were cultured in the above medium. The PGC#1 cell line was derived from a left frontal lobe IDH-wildtype glioblastoma (WHO IV), pathologically confirmed to meet diagnostic criteria for primary glioblastoma. Under microscopic observation, PGC#1 cells exhibit predominantly adherent growth, with a mixture of spindle-shaped, stellate, and polygonal cells. Some cells possess slender pseudopod-like protrusions, consistent with the malignant phenotype of high-grade glioma (Fig. S1D). In terms of growth characteristics, their proliferation rate is slower than that of immortalized cell lines (such as U251), with a population doubling time of approximately 48–72 h. Immunohistochemical profiling demonstrated positivity for GFAP, Oligo-2, Synaptophysin, Neu-N (neuronal marker), Vimentin, P53 (strongly positive, 80%; mutant type), EMA (focal positivity), S-100, CD34 (vascular staining), MGMT (90% positivity), Nestin, ATRX (90% retention), PTEN, H3K27Me3, and EGFR (+++ intensity), while showing negativity for CK, IDH-1, BRAF V600E, and H3K27M. Ki-67 proliferation index reached approximately 40% in hotspot areas, with preserved INI-1 expression. PGC#1 cells between passages 5–8 were utilized for subsequent experimental procedures. Cells were treated with different reagents according to research needs, including mevalonic acid (GLPBIO, Cat# 11668500, 100 μM, 48 h), simvastatin (APEBIO, Cat# A8522, 10 μM, 48 h), Ferrostatin-1 (MCE, Cat# HY-100579, 1 μM, 48 h), GPX4-IN-3 (MCE, Cat# HY-100579, 1 μM, 48 h), Erastin (MCE, Cat# HY-15763, 10 μM, 48 h), Liproxstatin-1 (MCE, Cat# HY-12726, 50 nM, 48 h), Z-VAD-FMK (MCE, Cat# HY-16658B, 40 μM, 48 h), Necrosulfonamide (MCE, Cat# HY-100573, 500 nM, 48 h). The above reagents were dissolved in dimethylsulfoxide (DMSO, Sigma-Aldrich, Cat# D2650; CAS: 67-68-5) (Table S1).

### Bioinformatics analysis

The CGGA database (http://www.cgga.org.cn/) was used to evaluate and analyse miR-139-5p expression levels in gliomas of different grades and tissue types and to analyse survival in patients with glioma. The TargetScanHuman 8.0 database (https://www.targetscan.org/vert_80/) was used to predict the gene-binding fragments between hsa-miR-139-5p and HMGCR in human cells.

### RNA extraction and qRT-PCR

The AG RNAex Pro extraction reagent (AGbio, AG21101, China) was used to extract total RNA from U251, U138, LN229 and PGC#1 cells. The concentration and purity of the extracted RNA were determined using a NanoDrop 2000 spectrophotometer (Thermo Fisher Scientific, Waltham, MA, USA) and then reverse-transcribed into stable cDNA using PrimeScript RT Master Mix (Takara Bio, RR036A, Shiga, Japan). The SYBR Green Pro Taq HS premixed qPCR Kit (AGbio, AG11740, China) was used for qRT-PCR in the CFX Connect System (Bio-Rad, Hercules, CA, USA) to analyse intracellular mRNA or miRNA expression levels, and the results were standardised to GAPDH and U6 expression. All experiments were independently repeated at least three times. The data were transformed and analysed using the △△CT method. GAPDH primer sequence (5′-3′): Forward ACTTCCTCTCTACAGTGCACGTG, Reverse TGCTGATGATCTTGAGGCTGTTGTC. hsa-miR-139-5p primer sequence (5′–3′): Forward ACTTCCTCTCTACAGTGCACGTG, Reverse ATCCAGTGCAGGGTCCGAGG, RT GTCGTATCCAGTGCAGGGTCCGAGGTATTCGCACTGGATACGACACTGGA.U6 primer sequence (5′–3′): Forward CTCGCTTCGGCAGCACA, Reverse AACGCTTCACGAATTTGCGT. HMGCR primer sequence (5′–3′): Forward ACAGATACTTGGGAATGCAGAG, Reverse CTGTCGGCGAATAGATACACC. SLC7A11 primer sequence (5′–3′): Forward TGTGTGGGGTCCTGTCACTA, Reverse CCTCCAAGAAGGGCCAGTTC. GPX4 primer sequence (5′–3′): Forward ACAAGAACGGCTGCGTGGTGAA, Reverse GCCACACACTTGTGGAGCTAGA.

### miR-139-5p overexpression or downregulation mediated by microRNA mimic or microRNA inhibitor

U251, U138, LN229 and PGC#1 cells were seeded onto the plate and incubated for 24 h until they attached to the bottom of the plate. The hsa-miR-139-5p mimic (gene sequence 5′–3′: sense UCUACAGUGCACGUGUCUCCAGU, antisense UGGAGACACGUGCACUGUAGAUU from Tsingke Biotech (Nanjing, China)) and hsa-miR-139-5p inhibitor (gene sequence 5′–3′: sense ACUGGAGACACGUGCACUGUAGA from Tsingke Biotech (Nanjing, China)) was used with the Lipofectamine 2000 Transfection Reagent (Invitrogen, 11668500) to transfect cells. The cells were transfected continuously for 72 h before the next experiment, and Lipofectamine 2000 was used as the vector for the control group. All operating procedures were performed in accordance with the manufacturer’s instructions.

### Cell transfection

hsa-miR-139-5p OE lentivirus (sequence: TCTACAGTGCACGTGTCTCCAGT) was synthesised by Tsingke Biotech (Nanjing, China). Lentivirus-transfected U251, U138 and PGC#1 cells, according to the manufacturer’s instructions, and then 2 μg/mL purinomycin screened for stable and high expression of miR-139-5p. The effects of lentiviral transfection were examined in various experiments.

### Western blot analysis

RIPA lysis buffer (CW2333S; CWBio) supplemented with 1% protease inhibitor (CW2200S; CWBio) and 1% phosphatase inhibitor (CW2383S; CWBio) was used to extract total protein from cells on ice. A bicinchoninic acid (BCA) assay kit (Beyotime, P0012, Beijing, China) was used to quantify the extracted proteins. After separation by sodium dodecyl sulphate-polyacrylamide gel electrophoresis, the protein extracts were transferred onto polyvinylidene fluoride (PVDF) membranes. The PVDF membrane was blocked in 5% skim milk at 26 °C for 1 h, incubated with the primary antibodies at 4 °C overnight, and finally incubated with the secondary antibodies at 26 °C for 1 h the next day. The membrane, covered with freshly made ECL solution (Millipore, WBKLS0500, USA), presented bands on an ImageQuant LAS500 chemiluminescence imager (General Electric, Boston, MA, USA). The values of the bands were analysed using ImageJ software (National Institutes of Health, USA). The primary antibodies used were anti-β-actin (Proteintech, 20536-1-AP, China, 1:1000), anti-GPX4 (Abcam, ab125066, USA, 1:2000), and anti-HMGCR (Abcam, ab242315, USA, 1:1000).

### Transmission electron microscopy (TEM)

The treated cells were collected and then fixed with glutaraldehyde. The cells were then soaked in 30%, 50%, 70%, and 100% alcohol for 10–20 min to dehydrate. Electron staining was performed with uranium acetate and lead citrate after embedding and slicing. The sample can then be viewed and photographed under a transmission electron microscope.

### RNA-seq analysis

The miR-139-5p mimic was transfected into U251 cells in a six-well plate for 72 h, and total RNA was extracted using TRIzol Reagent (Invitrogen, 15596026, USA) according to the manufacturer’s instructions. Total RNA was sent to IGE BIOTECHNOLOGY LTD (Guangzhou, China) for quality control, purification, screening, and transcriptome sequencing. Gene Set Enrichment Analysis (GSEA), Kyoto Encyclopaedia of Genes and Genomes (KEGG) pathway, and Gene Ontology (GO) analyses were used to analyse and present the results of RNA-seq (*p* < 0.05, for statistical significance).

### MDA assay

Malondialdehyde (MDA) content in cells transfected with miR-139-5p overexpressed lentivirus or treated with drugs was detected using an MDA content detection kit (Solarbio, BC0025, China) according to the manufacturer’s instructions. The specific operation steps were as follows: (1) the cells added with the special lytic solution for MDA extraction were broken by ultrasound, and then the supernatant was extracted after the broken cells were centrifuged at 8000 × *g* at 4 °C for 10 min. (2) The MDA detection reagent was prepared temporarily according to the manufacturer’s instructions, mixed with the supernatant, and heated in a 100 °C water bath for one hour. (3) The absorbance of the mixture at 532 nm and 600 nm was measured using a spectrophotometer, and the MDA content in the sample was calculated according to the formula provided by the manufacturer.

### GSH assay

A reduced glutathione (GSH) content assay kit (Solarbio, BC1175, China) was used to analyse the mean GSH content of cells under different treatments, as recommended by the manufacturer. GSH content was measured using a spectrophotometer at a wavelength adjusted to 412 nm and was calculated using a specific formula.

### GSSG assay

According to the instructions of the oxidized glutathione (GSSG) content detection kit (Solarbio, BC1185, China), 1 × 10⁶ cells were lysed in a lysis buffer containing a protein precipitant at a volume ratio of 1:5 (cells:lysis buffer). Cell lysis was achieved through either rapid freezing-thawing cycles in liquid nitrogen or ultrasonication (200 W, 3 s on/10 s off, 30 cycles). The lysate was then centrifuged at 12,000 × *g* for 10 min at 4 °C to obtain the supernatant. Subsequently, 2-vinylpyridine solution was added to block reduced glutathione (GSH). GSSG was enzymatically reduced to GSH by glutathione reductase. Following this, 5,5′-dithiobis-(2-nitrobenzoic acid) (DTNB) chromogenic reagent was added, and the absorbance change at 412 nm was dynamically monitored for 30–150 s. The GSSG content was calculated based on a standard curve.

### ROS and lipid peroxidation assay

To measure the level of intracellular Reactive oxygen species (ROS) in the different groups, DCFH-DA in the Reactive Oxygen Species Assay Kit (Solarbio, CA1410, China) was diluted 1:1000 and added to the collected cell suspension. And 10 μM BODIPY 581/591 C11 kit (KKL Med, KCM12619, USA) was used to detect lipid peroxidation. The cells were incubated at 37 °C for 20 min. The cells were washed with a serum-free medium to remove DCFH-DA or BODIPY 581/591 C11 that did not enter the cells. Finally, the fluorescence intensity of intracellular ROS (FITC) or lipid peroxidation (FITC) was measured using flow cytometry.

### Cholesterol assay

After the cells or tissues were treated with lentiviruses or drugs, they were collected and added to an extraction solution prepared according to the manufacturer’s instructions for the total cholesterol (TC) assay kit (Solarbio, BC1985, China) and then ultrasonically broken in an ice bath. Finally, the TC content in the cells or tissues was measured and calculated using a spectrophotometer and a standard curve.

### LC–MS/MS quantification of MVA and IPP

The sample pretreatment procedure involved rapid washing of cells with ice-cold PBS for cellular specimens, while tissue homogenate samples (50 mg) were snap-frozen in liquid nitrogen and subsequently homogenized. Metabolic processes were quenched using a pre-chilled methanol-water (1:1) system combined with ultrasonic fragmentation, followed by lipid removal through dichloromethane liquid-liquid extraction and final concentration via nitrogen evaporation. For derivatization, phosphate groups were specifically modified through a 30-min reaction at 40 °C using Tmt-PP reagent (12 mM) activated by EDC/HATU catalytic system. Chromatographic separation was performed on a BEH C18 column with a mobile phase gradient comprising aqueous solution containing 0.1% ammonium hydroxide and 10 mM ammonium acetate, along with 95% methanol-water system. Mass spectrometric analysis was conducted in positive ion mode, utilizing characteristic ion transition pairs for quantification: m/z 522.1 → 486.3 for MVA, while IPP was quantified through proportional analysis of m/z 619.8 → 454.4/552.0.

### Luciferase reporter system detects the activity of sec-tRNA

This study isolated total tRNA from differentially treated U251, U138, and PGC#1 cells, which was then co-incubated with wheat germ extract, recombinant human eEFSec, and SECIS-binding protein SBP2. To quantify selenocysteine insertion efficiency at UGA sites, we constructed a luciferase reporter vector (C258U-SECIS) containing both a UGA stop codon and the GPX4 3′UTR SECIS element, while using a UGC mutant vector as a nonspecific readthrough control. Luciferase activity was measured using the Dual-Luciferase Reporter Assay System (Promega) and normalized by Firefly/Renilla signal ratios. Comparative analysis of UGA codon readthrough efficiency across treatment groups provided an indirect assessment of sec-tRNA activity levels.

### Cell viability assay

Cells from different groups were seeded in 96-well plates at a number of 10^3^–10^4^ per well. After all cells were fully attached, the Cell Counting Kit-8 (CCK-8) (GLPBIO, GK10001, USA) was used to measure cell proliferation activity according to the manufacturer’s protocol. After the mixture of the CCK-8 reaction solution and cells was incubated in a humid incubator for 1–4 h, a spectrophotometer adjusted to the absorbance at 450 nm was used to analyse the cell viability.

### TUNEL assay

Rapid One Step TUNEL Apoptosis Assay Kit (KeyGen Biotech, Cat# KGA1408-20) was used to detect cell apoptosis. A certain number of cells were planted on the plate and fixed at room temperature for 30 min with 4% paraformaldehyde. The cells were rinsed three times with PBS and then permeated with 1%Triton X-100. Finally, TUNEL reaction solution was prepared according to the instructions and added to the cells and incubated at 37 °C away from light for 1 h. The apoptosis-related fluorescence was observed under fluorescence microscope (TexaRed).

### Steroid metabolomics analysis

The cells were transferred into a centrifuge tube after washing with PBS solution 2–3 times. The cells were then collected by centrifugation at 300–500 × *g*. Finally, the cells were sent to Metware Biotechnology Inc (Wuhan, China) for subsequent processing and LC–MS analysis.

### Fe^2+^ assay

The ferrous ion content detection kit (Solarbio, BC5415, China) utilizes a chemical colorimetric method. An appropriate amount of cell sample was taken, and the cell count to extract volume ratio was maintained at 1:1 (e.g., 2 × 10^7^ cells with 1 mL of extract). The sample was then subjected to ice-bath sonication (200 W power, 5 s on/5 s off interval, for 5 min). Following centrifugation (10,000 × *g*, 4 °C, 10 min), the supernatant was collected. Tripyridyltriazine chromogenic reagent was added, and the reaction was allowed to proceed at 37 °C for 10 min. The reaction mixture was then transferred to a 96-well plate, and the absorbance was measured at 593 nm using a microplate reader. Quantification was achieved by comparing with a standard curve.

### Animal experiment

Fifty thymus-free male BALB/c nude mice aged 3–5 weeks were obtained from GemPharmatech Co., Ltd., raised in a pathogen-free environment, and provided with 12-h day–night cycle and free access to food and water. To investigate the effects of miR-139-5p and SIM on tumour progression and cholesterol biosynthesis in vivo. Mice were randomly divided into four groups (*n* = 10 per group). (1) Negative control group: mCherry-LUC Vector-transfected U251 cells were injected orthotopically into the right hemisphere of the mouse brains. Besides, U251 cells in the logarithmic growth phase were evenly dispersed in 100 μL PBS and injected subcutaneously into the right side of the mouse trunk to establish a glioma animal model. Three days after the cells were injected into the brains or after approximately 20 days, the tumour size reached about 150 mm^3^, and the mice were intraperitoneally injected with the vector. (2) miR-139-5p OE group: U251 cells transfected with miR-139-5p overexpressing lentivirus were injected into the mouse brains or under the right trunk of the mice according to the above method. They were also intraperitoneally injected with the vector after three days injecting cells into the brains or when the subcutaneous tumour size increased to 100–150 mm^3^. (3) SIM group: The mouse glioma model was constructed according to the above method, and 10 mg/kg SIM was intraperitoneally injected once every three days after three days injecting cells into the brains, for five administrations. (4) miR-139-5p OE + MVA group: The mouse U251 miR-139-5p OE glioma model was constructed according to the above method, and 100 mg/kg MVA was injected intraperitoneally once every three days after three days injecting cells into the brains or when the subcutaneous tumour size increased to 100–150 mm^3^, for five administrations. (5) miR-139-5p OE + SIM group: The mouse U251 miR-139-5p OE glioma model was constructed according to the above method, and 10 mg/kg SIM was intraperitoneally injected once every three days after three days injecting cells into the brains or when the subcutaneous tumour size increased to 100–150 mm^3^, for five administrations. After 15 days, all mice were euthanised under anaesthesia with phenobarbital sodium at a dose of 100–120 mg/kg, and the subcutaneous tumour tissue was dissected. Tumour size was measured with the specimen fixed measuring plate and calculated using the following formula: tumour size = length × width × 0.5. Tumour weight was measured using a weighing scale. The total cholesterol content of the tumour tissue was determined using a cholesterol-measuring kit (Solarbio, Cat# BC1985). The Kaplan–Meier method was used to calculate the overall survival of the mice. The size of the brain tumour was measured in vivo imaging device (IVIS Lumina II, USA). All experimental procedures were approved by the Animal Ethics Committee of Southern Medical University in accordance with the guidelines of the Institutional Animal Care and Use Committee (Project No. LAEC-2023-114).

### Statistical analysis

All experimental data were collected and analysed using GraphPad Prism 9.0 Software (GraphPad Software, Boston, MA, USA). Results are presented as the mean ± standard error of the mean (SEM). Each experiment was independently repeated at least three times. Statistical significance of the experimental results for the two groups was calculated using an unpaired two-tailed Student’s *t*-test. We used ANOVA to calculate the statistical significance of multiple experimental results. Statistical significance of the survival curves was determined using the log-rank test. Statistical significance was defined as *p* < 0.05.

## Supplementary information

Supplementary Table S1

Supplementary Figures and Figure legends

uncropped blots

## Data Availability

The authors declare that all experimental results of this study can be replicated under correct guidance.
