# Supplementary material for: miR-139-5p activates ferroptosis by inhibiting the expression of HMG-CoA reductase to inhibit the progression of glioma
Source: Cell Death Discov. 2025 May 21;11:245. doi: 10.1038/s41420-025-02532-7 (PMC12095534; doi:10.1038/s41420-025-02532-7)
Supplement: Supplementary file 2 — Supplementary Figures and Figure legends [file 41420_2025_2532_MOESM2_ESM.pdf]

Supplementary Figures and Figure legends

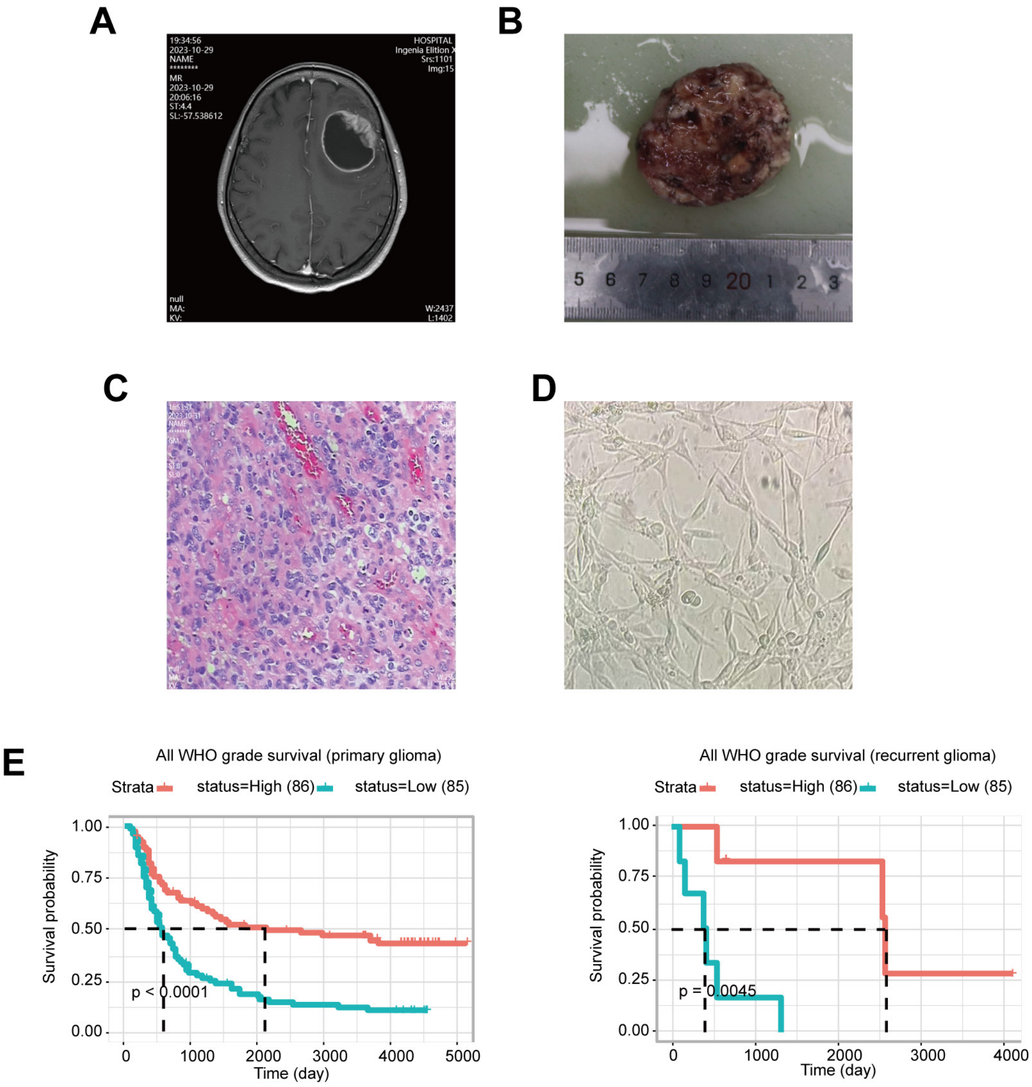

Supplementary Figure 1. Gliomas with low miR-139-5p expression predict a worse prognosis: (A-C) The MRI, macroscopic gross view and representative H&E staining images of the glioma sample from which the primary glioma cells (PGC#1) originated. (D) The morphology of PGC#1 cells under the microscope. (E) Survival analysis of the CGGA database indicated that the lower the expression level of miR-139-5p, the worse the prognosis of the first and recurrent glioma patients. Data are presented as mean ± standard deviation (SD) from three independent experiments. ***$p < 0.001$, **$p < 0.01$, *$p < 0.05$.

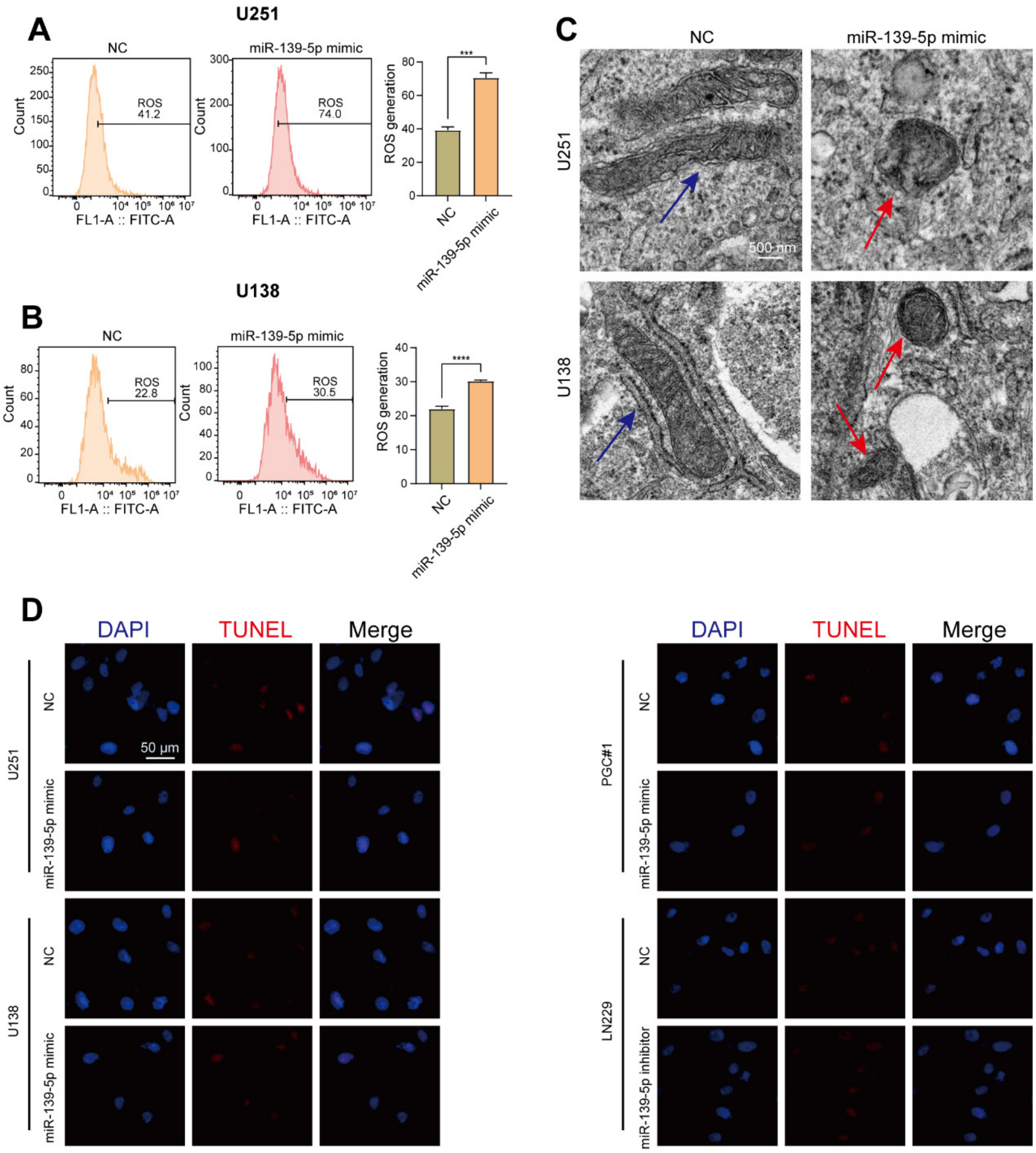

Supplementary Figure 2. Overexpression of miR-139-5p promotes ferroptosis in glioma cells: (A, B) Flow cytometry histogram showing that the ROS level of the miR-139-5p mimic group in U251 and U138 cells was higher than that in the NC group. (C) Transmission electron microscopy (TEM) was used to observe the morphological changes of mitochondria in U251 and U138 cells after treatment. (D) TUNEL staining was used to detect apoptosis levels. Data are presented as mean ± standard deviation (SD) from three independent experiments. ***$p < 0.001$, **$p < 0.01$, *$p < 0.05$.

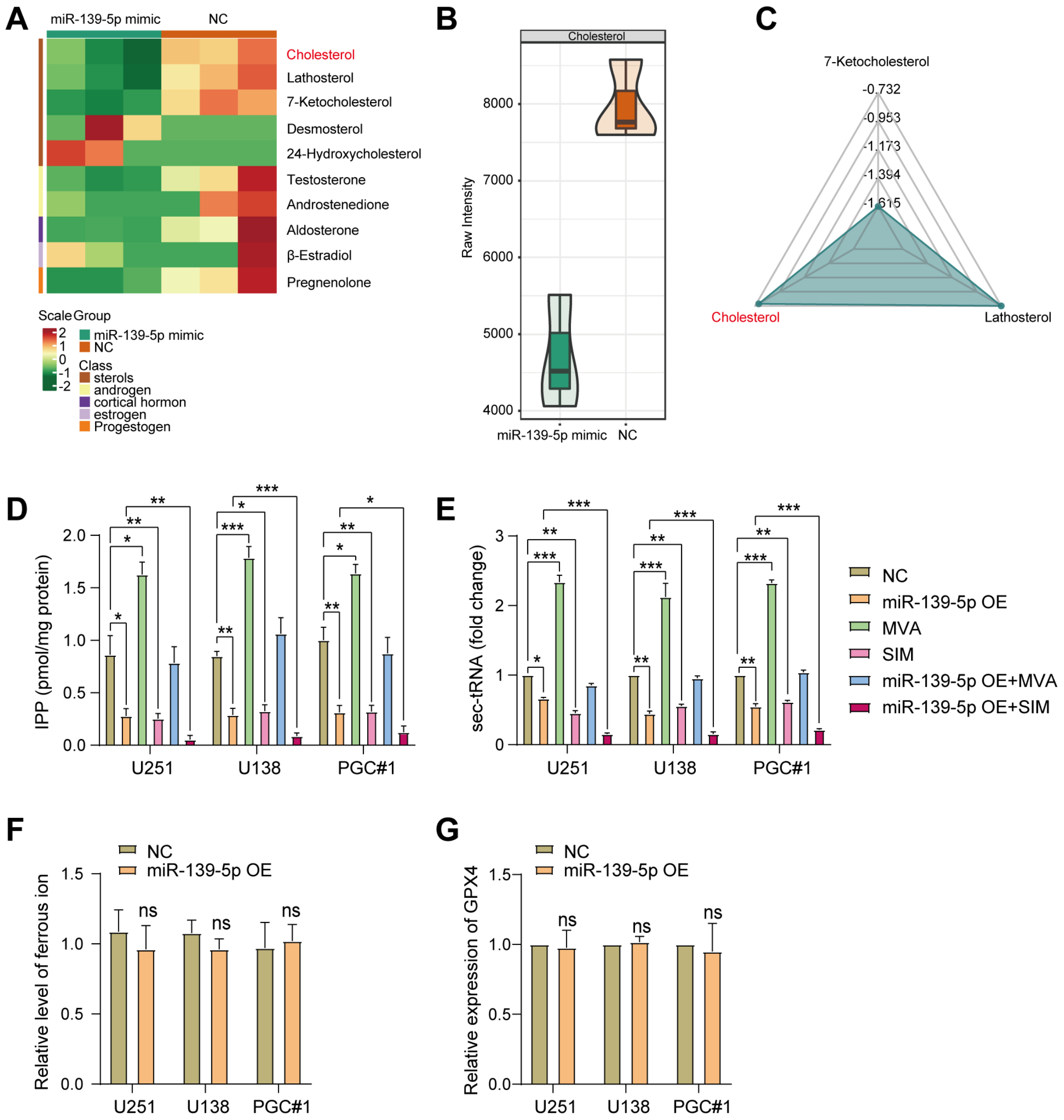

Supplementary Figure 3. miR-139-5p inhibits the mevalonate pathway in glioma cells: (A-C) Steroid metabolomics analysis was used to detect the changes in cholesterol-related metabolism after miR-139-5p overexpression. (D) LC-MS/MS was employed to quantitatively analyze the intracellular levels of isopentenyl pyrophosphate (IPP). (E) The luciferase reporter system was used to detect sec-tRNA activity. (F) The relevant kit was used to detect the content of ferrous ions in the cells. (G) qRT-PCR was used to assess the mRNA expression levels of GPX4 in the cells. Data are presented as mean ± standard deviation (SD) from three independent experiments. ***$p < 0.001$, **$p < 0.01$, *$p < 0.05$.

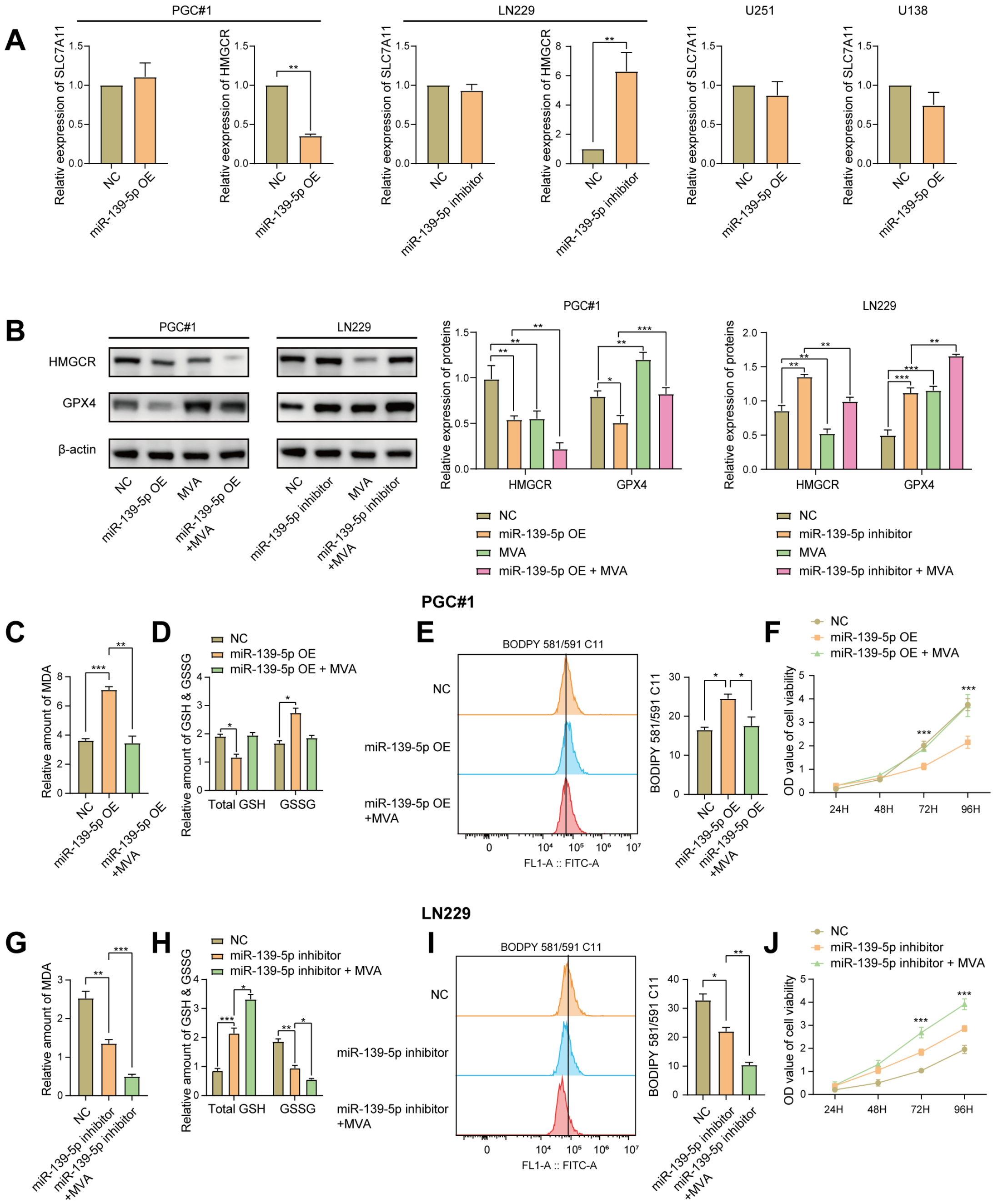

Supplementary Figure 4. miR-139-5p promotes ferroptosis by inhibiting the HMGCR expression: (A) qRT-PCR assay revealed that the expression of SLC7A11 and HMGCR in cells transfected with miR-139-5p overexpressed lentivirus or treated with miR-139-5p inhibitor. (B) Western blotting was used to determine the expression levels of related proteins in cells under various treatment conditions. Results of western blotting of PGC#1 and LN229 cells were statistically analysed. (C, D, G, H) The relevant kit was used to detect MDA, GSH & GSSG levels in the PGC#1 and LN229 cells. (E, I) Flow cytometry was used to detect the lipid peroxidation in cells. Statistical results of lipid peroxidation level in cells were analysed. (F, J) Proliferative activity of cells was analysed using the CCK-8 assay. Data are presented as mean ± standard deviation (SD)

from three independent experiments. ***$p < 0.001$, **$p < 0.01$, *$p < 0.05$.

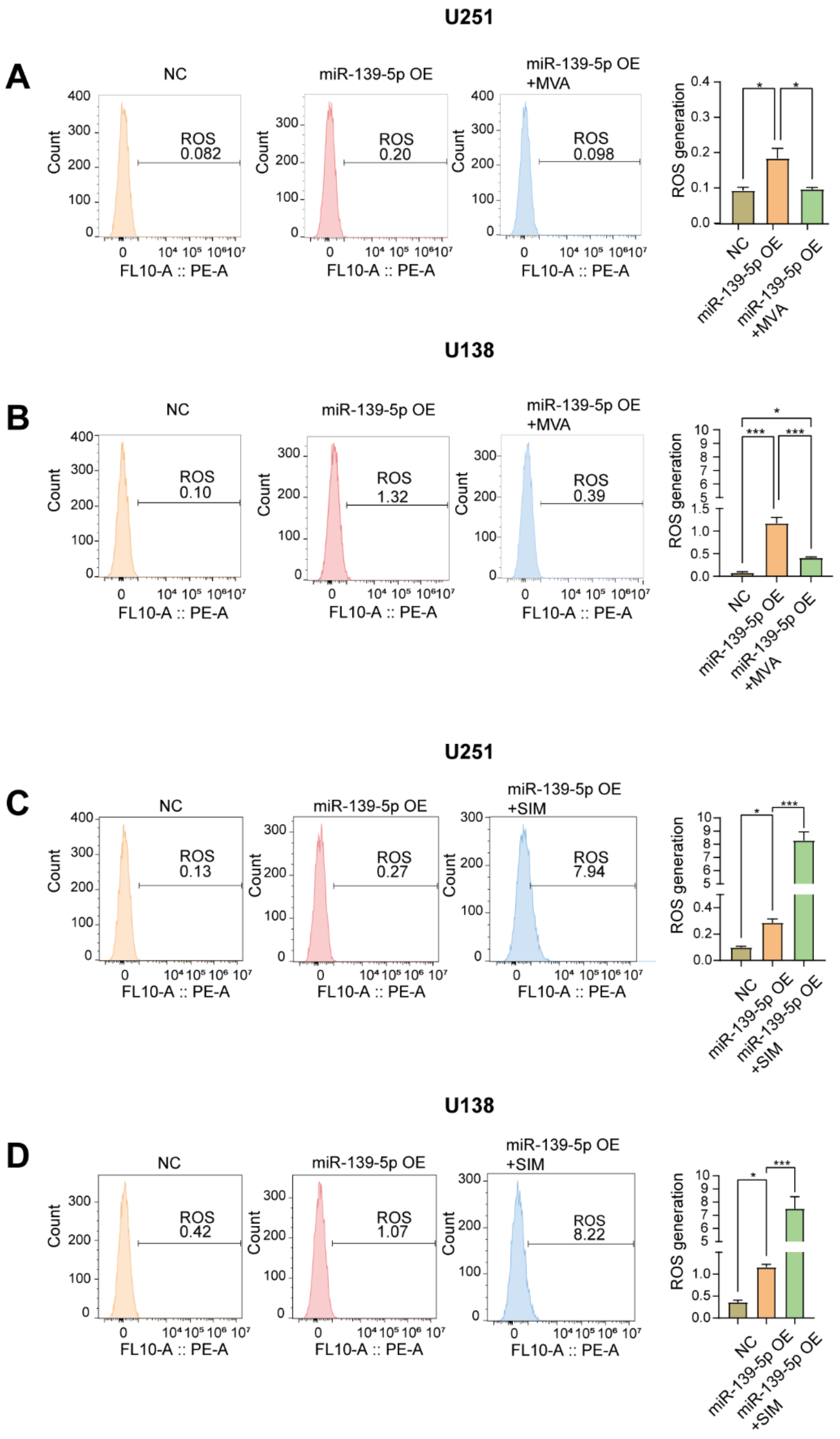

Supplementary Figure 5. miR-139-5p synergistically promotes ferroptosis with simvastatin by inhibiting the HMGCR expression: (A-D) Flow cytometry was used to detect the ROS levels in U251 and U138 cells. Statistical results of intracellular ROS level in cells were analysed. Data are presented as mean ± standard deviation (SD) from

three independent experiments. ***$p < 0.001$, **$p < 0.01$, *$p < 0.05$.

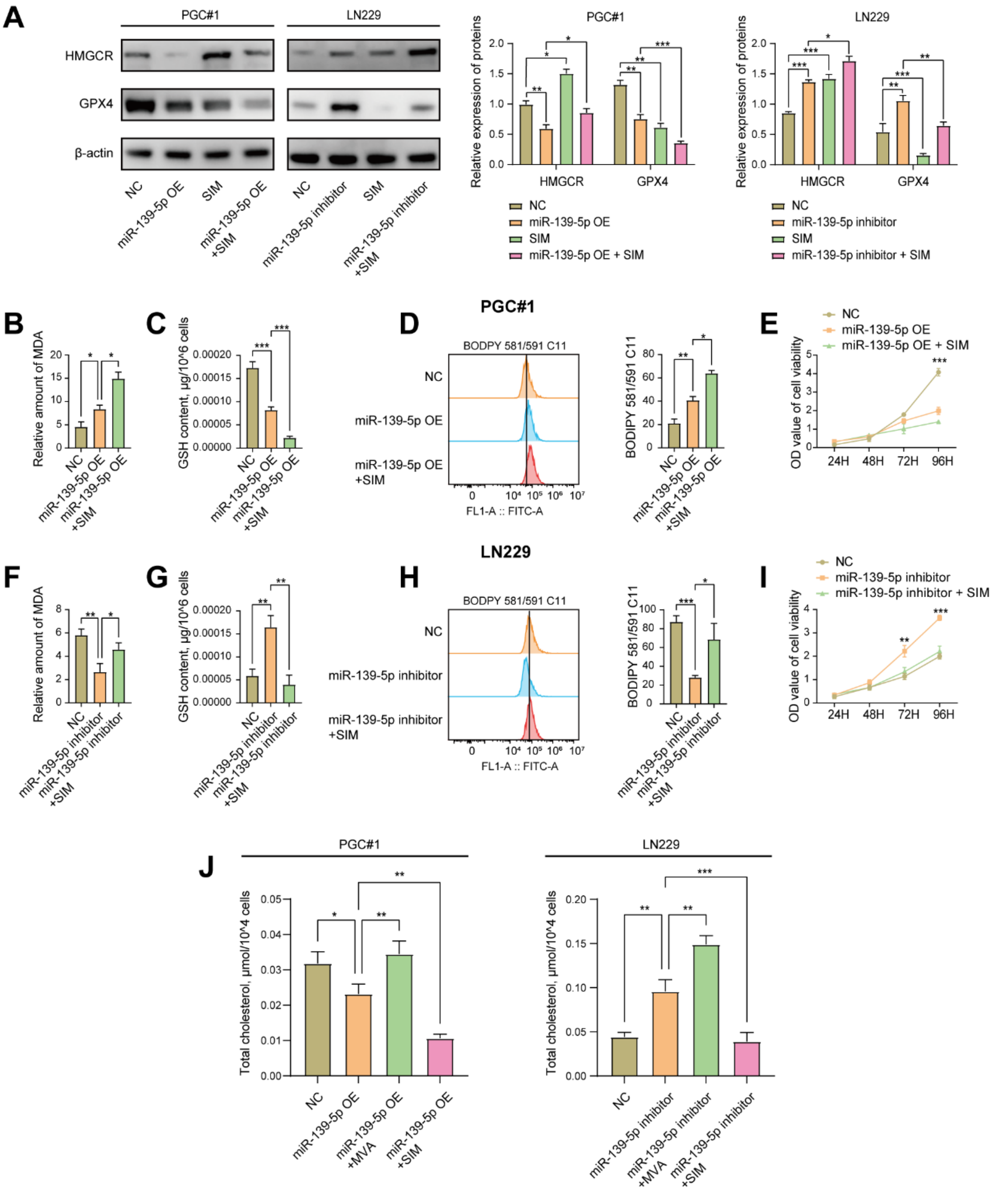

Supplementary Figure 6. miR-139-5p synergistically promotes ferroptosis and inhibits cholesterol synthesis with simvastatin: (A) Western blotting was used to determine the expression levels of related proteins in cells under various treatment conditions. Results of western blotting of PGC#1 and LN229 cells were statistically analysed. (B, C, F, G) The relevant kit was used to detect MDA, GSH & GSSG levels in the PGC#1 and LN229 cells. (D, H) Flow cytometry was used to detect the lipid peroxidation levels in PGC#1 and LN229 cells. Statistical results of lipid peroxidation level in cells were analysed. (E, I) Proliferative activity of PGC#1 and LN229 cells was analysed using the CCK-8 assay. (J) The relevant kit was used to detect cholesterol

levels in the PGC#1 and LN229 cells. Data are presented as mean ± standard deviation (SD) from three independent experiments. ***$p < 0.001$, **$p < 0.01$, *$p < 0.05$.

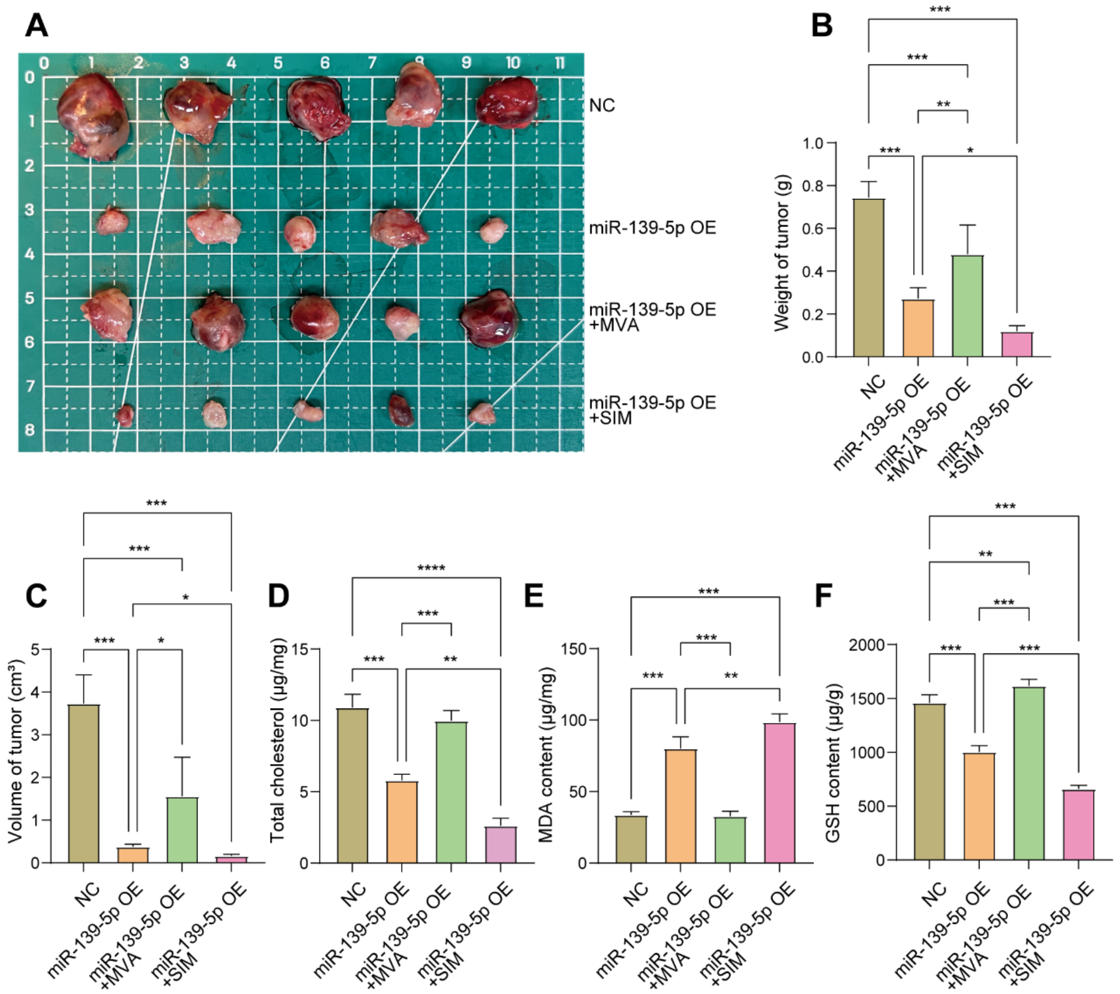

Supplementary Figure 7. miR-139-5p promotes ferroptosis and inhibits cholesterol biosynthesis and tumour progression *in vivo*: (A) U251 cells or U251 miR-139-5p OE cells were injected subcutaneously into Balb/c nude mice, and then 100 mg/kg MVA or 10 mg/kg SIM were injected intraperitoneally every three days to treat mice in the miR-139-5p OE group, for five times. Tumour anatomical results suggested that overexpression of miR-139-5p inhibited the proliferation of glioma *in vivo*, MVA reversed the suppression of tumour progression by overexpression of miR-139-5p, SIM and overexpression of miR-139-5p synergistically inhibited tumour proliferation. (B) Statistical results of subcutaneous tumour weights in BALB/c nude mice. (C) Statistical results of the subcutaneous tumour volume in BALB/c nude mice. (D) The total cholesterol content of the tumour tissues was detected using the cholesterol kit. These results align with *in vitro* experiments. (E) MDA kit was used to detect the content of MDA in tumour tissues. (F) The GSH content in tumour tissues was detected by GSH kit. (G) The results of the Balb/c nude mouse survival analysis were consistent with those of the *in vitro* experiments. Data are presented as mean ± standard deviation (SD)

from three independent experiments. ***$p < 0.001$, **$p < 0.01$, *$p < 0.05$.
