# Supplementary figures and images for: miR-139-5p activates ferroptosis by inhibiting the expression of HMG-CoA reductase to inhibit the progression of glioma
Source: Cell Death Discov. 2025 May 21;11:245. doi: 10.1038/s41420-025-02532-7 (PMC12095534; doi:10.1038/s41420-025-02532-7)

**Figure 4F**

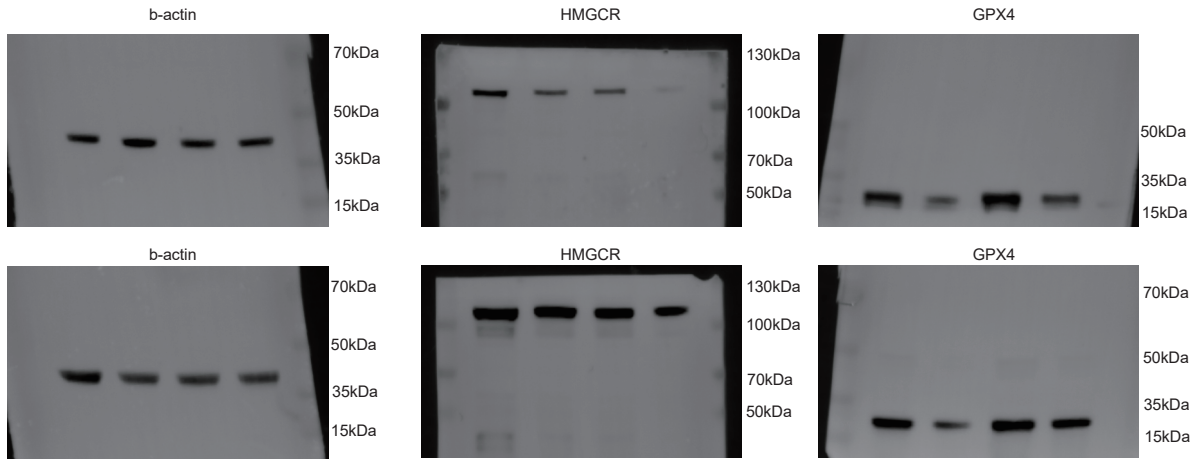

**Figure S4B**

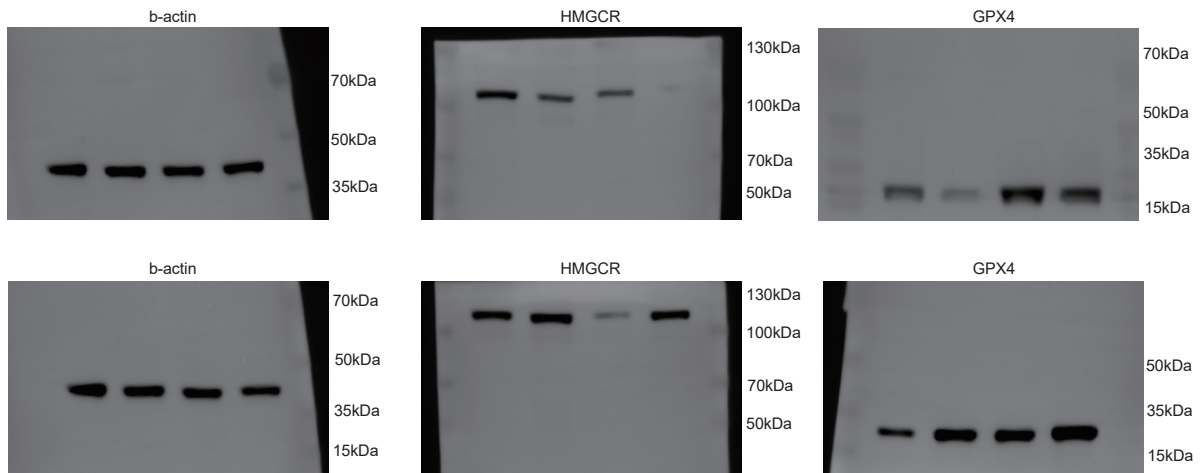

**Figure 5A**

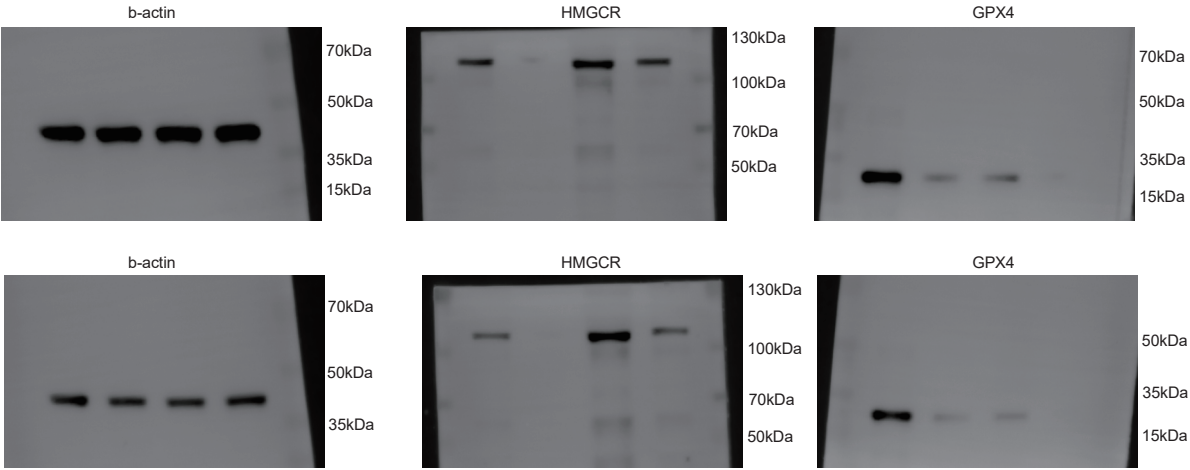

**Figure S6A**

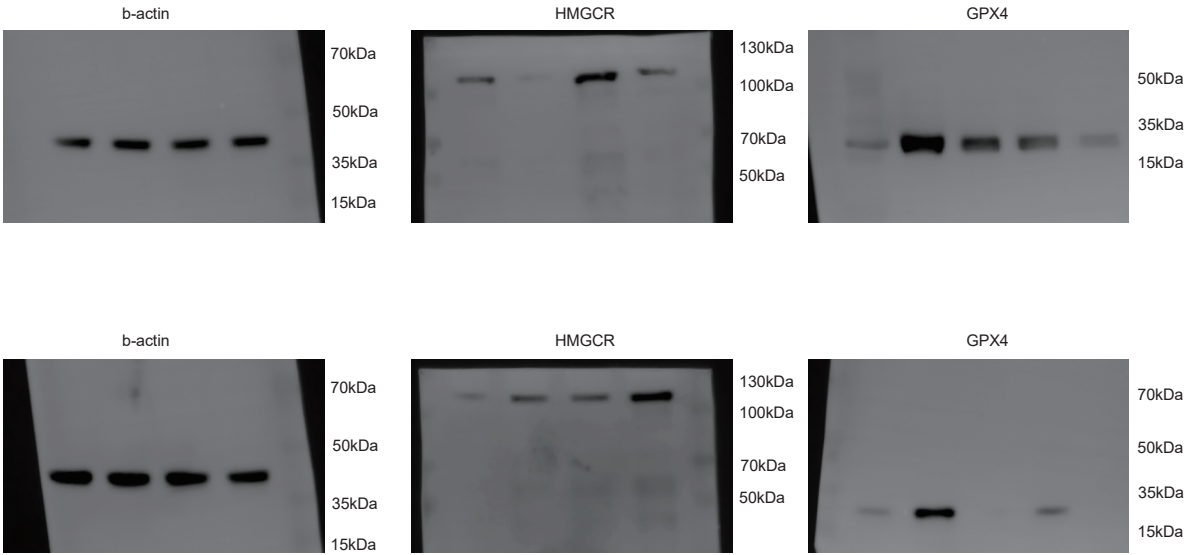

Supplement: Supplementary file 3 — uncropped blots [file 41420_2025_2532_MOESM3_ESM.pdf]
